# DNA Nanomachine (DNM) Biplex Assay for Differentiating *Bacillus cereus* Species

**DOI:** 10.3390/ijms24054473

**Published:** 2023-02-24

**Authors:** Muhannad Ateiah, Erik R. Gandalipov, Aleksandr A. Rubel, Maria S. Rubel, Dmitry M. Kolpashchikov

**Affiliations:** 1 Laboratory of Solution Chemistry of Advanced Materials and Technologies, ITMO University, Lomonosova St. 9, St. Petersburg 191002, Russia; muhannad@scamt-itmo.ru (M.A.); gandalipov@scamt-itmo.ru (E.R.G.); rubel@scamt-itmo.ru (M.S.R.); 2 Laboratory of Amyloid Biology, St. Petersburg State University, Universitetskaya enb. 7-9, St. Petersburg 199034, Russia; a.rubel@spbu.ru; 3 Chemistry Department, University of Central Florida, 4000 Central Florida Boulevard, Orlando, FL 32816-2366, USA; 4 Burnett School of Biomedical Sciences, University of Central Florida, Orlando, FL 32816, USA

**Keywords:** *B. cereus*, detection of folded RNA, 10–23 DNAzyme, amplification-free detection, binary probes, 16S rRNA, single nucleotide selectivity

## Abstract

Conventional methods for the detection and differentiation of *Bacillus cereus* group species have drawbacks mostly due to the complexity of genetic discrimination between the *Bacillus cereus* species. Here, we describe a simple and straightforward assay based on the detected unamplified bacterial 16S rRNA by DNA nanomachine (DNM). The assay uses a universal fluorescent reporter and four all-DNA binding fragments, three of which are responsible for “opening up” the folded rRNA while the fourth stand is responsible for detecting single nucleotide variation (SNV) with high selectivity. Binding of the DNM to 16S rRNA results in the formation of the 10–23 deoxyribozyme catalytic core that cleaves the fluorescent reporter and produces a signal, which is amplified over time due to catalytic turnover. This developed biplex assay enables the detection of *B. thuringiensis* 16S rRNA at fluorescein and *B. mycoides* at Cy5 channels with a limit of detection of 30 × 10^3^ and 35 × 10^3^ CFU/mL, respectively, after 1.5 h with a hands-on time of ~10 min. The new assay may simplify the analysis of biological RNA samples and might be useful for environmental monitoring as a simple and inexpensive alternative to amplification-based nucleic acid analysis. The DNM proposed here may become an advantageous tool for detecting SNV in clinically significant DNA or RNA samples and can easily differentiate SNV under broadly variable experimental conditions and without prior amplification.

## 1. Introduction

Among many bacteria, the *Bacillus cereus* group stands out for its diversity and ubiquitous spread along the biosphere. Certain species are pathogenic with foodborne and infectious potential [1,2,3]. For instance, *Bacillus cereus* (*B. cereus*) is a well-recognized pathogen that causes two different forms of food poisoning, including emesis and diarrhea [4]. In contrast to *B. cereus*, *Bacillus thuringiensis* (*B. thuringiensis*) is categorized as non-harmful for humans and exploited as a pesticide targeting insects [5,6,7]. Nonetheless, *B. thuringiensis* has also been reported in the context of foodborne outbreaks [8]. *B. cereus*, *B. thuringiensis*, *Bacillus anthracis*, and *Bacillus mycoides* (*B. mycoides*) are classified collectively as members of the *B. cereus* group based on their close similarity. The actual frequency of *B. thuringiensis* in foodborne outbreaks is still unknown due to the complexity of the genetic discrimination between the *Bacillus cereus* species. Genomes of *B. cereus*, *B. thuringiensis*, and *B. mycoides* are especially infused with each other [9,10,11].

The main method of *B. thuringiensis* discrimination uses *cry* genes [6,12]. However, this approach is hampered by a high number of polymorphisms [13,14,15], both by some *B. cereus* strains containing *cry*-like genes [16] and by the discovery of some *cry* genes in different *Bacillus* species or even in other genera [17,18]. The two species exhibit high resemblance in biochemical results [19] and genetic properties [20,21]. Furthermore, Pfrunder et al. [22] demonstrated that standard biotyping cannot be used to accurately identify species of the *B. cereus* group. To achieve effective discrimination, some complex and elaborate techniques were developed including two-step LAMP [23], as well as sensitive detection of the pathogens via aptamers in association with magnetic and upconversion nanoparticles [24] or to xMAP technology [25]. These techniques require specific conditions and equipment often lacking in medical facilities. Because of this, biochemical tests might not be sufficient for differentiation [22]. To date, there is no well-established approach in routine diagnostics to discriminate pathogen origin and so the International Organization for Standardization (ISO) guidelines presume all the *B. cereus* group outbreaks to be of *B. cereus* origin (ISO 7932:2004). The problem of *Bacillus cereus* species group determination requires new tools for rapid, easy-to-use, cheap, and, at the same time, SNV-sensitive detection that can be applied later to other subjects.

Nucleic acid amplification–based tests are the golden standard of molecular diagnostics [26,27,28]. Isothermal amplification techniques are point-of-care (POC) compatible, since they do not require the expensive thermocyclers that are needed for the most reputable polymerase chain reaction (PCR)–based diagnostics [29,30]. Often, hybridization probes are used to report the presence of a specific nucleic acid [31,32,33]. However, the formation of a complex between a probe and a long single stranded (ss) DNA or RNA analyte is complicated by the analyte’s secondary and tertiary structures, which are especially stable in the case of RNA [34,35]. A traditional approach to overcome the problem is to target opened RNA fragments, which are not involved in structures predicted by the folding software [36]. However, the search for accessible RNA fragments requires labor-intensive trial-and-error experiments since computational prediction of RNA folding is still under development [37]. Other approaches to addressing this challenge include using a structure-free DNA with pseudo-complementary properties [38], or LNA- and PNA-containing oligonucleotides that form thermodynamically more stable complexes than natural nucleic acids and thus can invade the natural hybrids [39,40]. However, LNA and PNA are expensive and demonstrate low selectivity in binding complementary nucleic acids [41].

**Figure 1:**
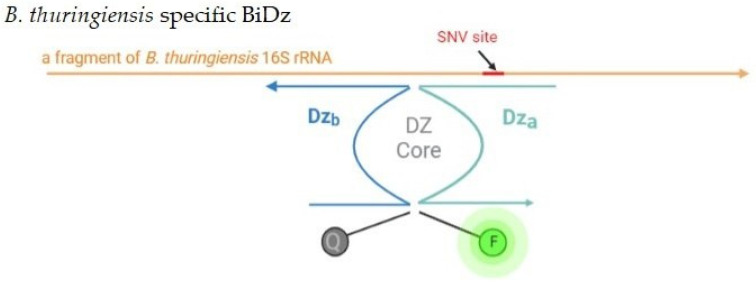
Design of BiDz probes used in this study. Design of *B. thuringiensis*–specific BiDz probe. DNA strand Dz_a_ and Dz_b_ bind RNA analyte and form a catalytic core that cleaves fluorophore and quencher labelled F-sub. The design of *B. mycoides*–specific BiDz probe differs from the design of *B. thuringiensis*–specific BiDz probe in the reporter substrate used. The Cy-sub reporter substrate was used with the *B. mycoides*–specific BiDz probe. The difference between F-sub and Cy-sub sequence is shown in Table S1.

Protein-free assays that utilizes all-DNA RNA-cleaving deoxyribozymes (Dz) for signal amplification have been developed [42,43,44]. For diagnostic purposes, a Dz can be split into two fragments to form a binary Dz (BiDz) sensor (Figure 1) [42,43]. BiDz_a_ and BDz_b_ hybridize to a complementary fragment in nucleic acid analyte and form a catalytically active Dz core, which can cleave a specifically designed fluorophore and quencher labeled reporter substrate (F-sub or Cy-sub in Figure 1), thus producing a fluorescent output. The signal is accumulated over time due to the catalytic turnover. BiDz sensors demonstrate improved sensitivity over other hybridization probes (e.g., molecular beacon and adjacent probes) due to the catalytic amplification. It was shown that under optimized assay conditions, BiDz based of 10–23 has k_cat_ 80 min^−1^ [43], thus providing the upper limit for an amplification factor of 4800 in one hour under the condition of 100% complex formation with the analyte, fast F-sub delivery. Another BiDz advantage is its excellent selectivity in differentiating single-base substitutions, which is attributed to its split design [45].

Our continuing efforts [46,47,48,49] in perfecting BiDz for the detection of nucleic acids led to the BiDz-base nanostructure, the so-called “DNA nanomachine” (DNM) [34]. The DNM is equipped with two more analyte-binding arms attached to the common dsDNA scaffold [34,50]. Such a structure was proven to enhance the sensitivity of the conventional BiDz and helped unwind the target nucleic acid due to the improved binding affinity of the arms [45,51,52] and through displacing the complementary strand of dsDNA or fragments of the complex secondary structure of RNA. Unwinding of the target nucleic acid facilitated the formation of a signal-producing complex in an increased concentration, thus reducing the time of the assay and enhancing the fluorescent signal.

**Figure 2:**
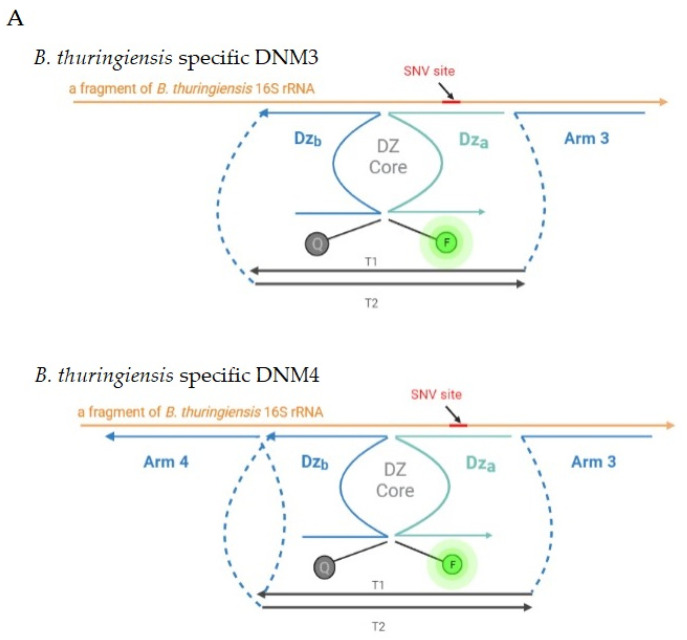

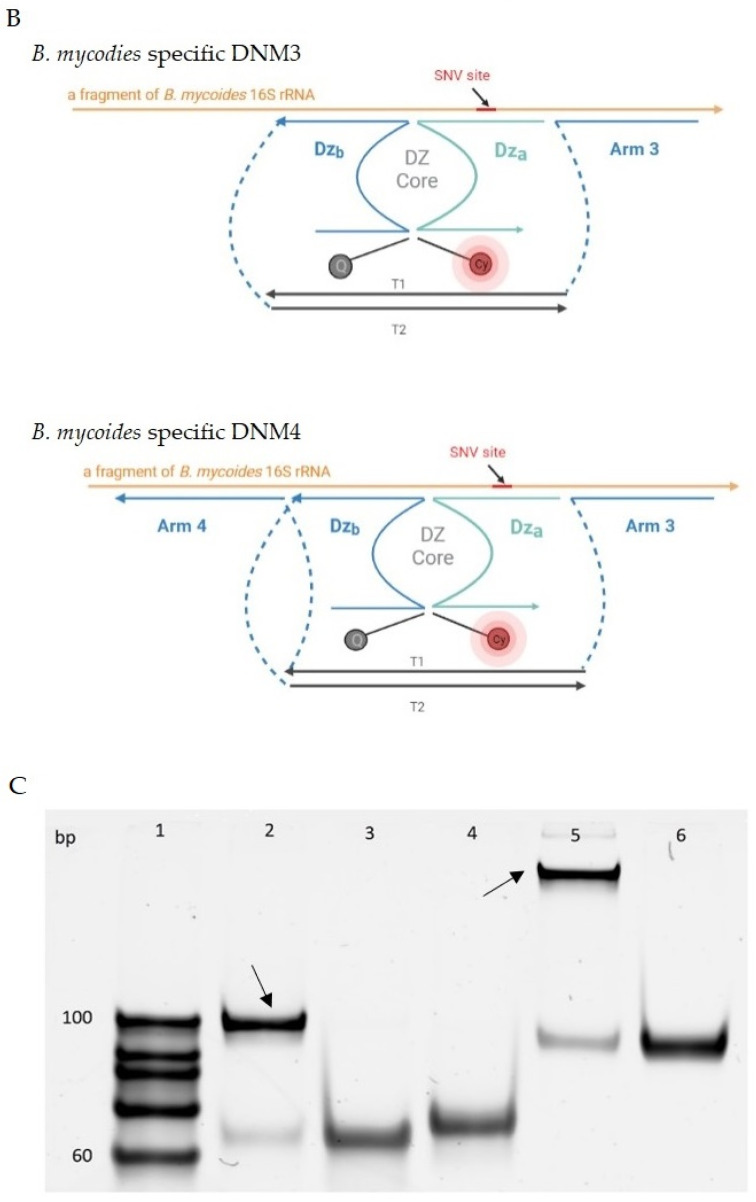
Design of DNM3 and DNM4 used in the study. (**A**) Design of *B. thuringiensis*–specific DNM3 and DNM4. DNM3 has a separate arm (Dz_a_) and two arms (Dz_a_ and Arm 3) attached to the dsDNA scaffold (T1 and T2). DNM4 has a separate arm (Dz_a_) and three arms (Dz_a_, Arm 3 and Arm 4) attached to the dsDNA scaffold. Arms 3 and 4 were designed to tightly bind 16S rRNA, thereby unfolding its secondary structure. DNM3 and 4 cleaved F-sub, producing fluorescent output at 525 nm. (**B**) Design of *B. mycoides*–specific DNM3 and DNM4 was accomplished similar to that of DNMs for *B. thuringiensis*, but it cleaved Cy-Sub, producing fluorescent output at 617 nm. (**C**) Analysis of DNMs’ associations in PAGE gel: 1—Ladder; 2—DNM3 (T1, T2 strands annealed); 3—Tile strand T1; 4—T2 strand; 5—DNM4 (T1, T2′ stands annealed); 6—Tile T2′ strand (see Table S1 for sequences). The correct assembly of DNM3 and DNM4 are indicated by the arrows.

In this study, we demonstrate the general applicability of the DNM approach in targeting and SNV discrimination in another scoop of nucleic acids, natural 16S rRNA without prior amplification. We demonstrate the ability of the DNM approach to outcompete the intramolecular base pairing of the RNA target and to “open up” the targeted fragment. We developed six variations of DNA nanosensors with a different number of DNA-binding arms for the detection and differentiation of *B. thuringiensis* and *B. mycoides*. For each strain, three DNA nanosensors were developed: BiDz sensor (Figure 1), three-armed DNA nanomachine (*B. thuringiensis*-specific DNM3 and *B. mycoides*-specific DNM3, in Figure 2A,B, top), and four-armed DNA nanomachine (*B. thuringiensis*-specific DNM4 and *B. mycoides*-specific DNM4, in Figure 2A,B, bottom), attributable to one or two additional unwinding arms in comparison to the BiDz sensor, respectively. Two variants of the reporter substrate were used: F-sub was used for detecting *B. thuringiensis* and Cy-sub was used for *B. mycoides* (see Table S1 for sequences).

## 2. Results

### 2.1. Design of DNA-Nanosensors and Preliminary Efficiency Tests

Initially, BiDz sensors based on 10–23 deoxyribozyme were used, an analogue to that used earlier for detection of *E. coli* cells [45], quantification of mutant ribosomal RNA [48], and analysis of *Mycobacterium tuberculosis* RNA [53]. The sequences of Dz_a_, Dz_b_, and the reporter substrates used in this study are listed in Table S1. Two BiDz sensors targeting *B. thuringiensis* (Figure 2) and *B. mycoides* 16S rRNA were designed. The sequences of 16S rRNA were obtained from the work of Ash et al. [54] and aligned (Figure S1). The sensor *B. thuringiensis*–specific BiDz was complementary to *B. thuringiensis* 16S rRNA (position 172–203). The sensor *B. mycoides*–specific BiDz was complementary to *B. mycoides* 16S rRNA (position 169–206). The design of the sensors differs in the targeted SNV and the reporter that was used. Strands *B. thuringiensis*–specific Dz_a_ and *B. mycoides*–specific Dz_a_ were complementary to a fragment of 16S rRNA containing two SNVs (192 and 197), which are shown in bold sequence in Table 1.

SNV 197 was used to differentiate between *B. thuringiensis* and *B. mycoides*, whereas SNS 192 was used to differentiate between two strains from *B. cereus* and *Bacillus anthracis*. The analyte-binding arm of the Dz_a_ strand was designed to be short enough to form a stable hybrid only with the specific target, thus enabling the high selectivity of 16S rRNA recognition, according to the fundamental principles of the binary probe design [45]. The sensors were tested using the total RNA extracted from *B. thuringiensis* and *B. mycoides* (Figure S1) and did not show any response above the background signal, both after 1 h (Figure in Section 2.4) and after 3 h of incubation (Figures S2 and S3). This can be attributed to the fact that the RNA region complementary to *B. thuringiensis*–specific BiDz and *B. mycoides*–specific BiDz is involved in a stable stem-loop structure (Figure S4).

To access the SNV site within the tight 16S rRNA structure, we designed the three- and four-armed DNM3 and DNM4, respectively. The *B. thuringiensis*–specific DNM4 is a four-armed machine, complementary to *B. thuringiensis* 16S rRNA (position 143–232) and *B. thuringiensis*–specific DNM3-thu, a three-armed machine for the same region. The *B. mycoides*–specific DNM4 is a four-armed nanomachine, complementary to *B. mycoides* 16S rRNA (position 143–232) and *B. mycoides*–specific DNM3, a three-armed machine complementary for the same region.

### 2.2. Characterization of BiDzs and DNMs

**Figure 3:**
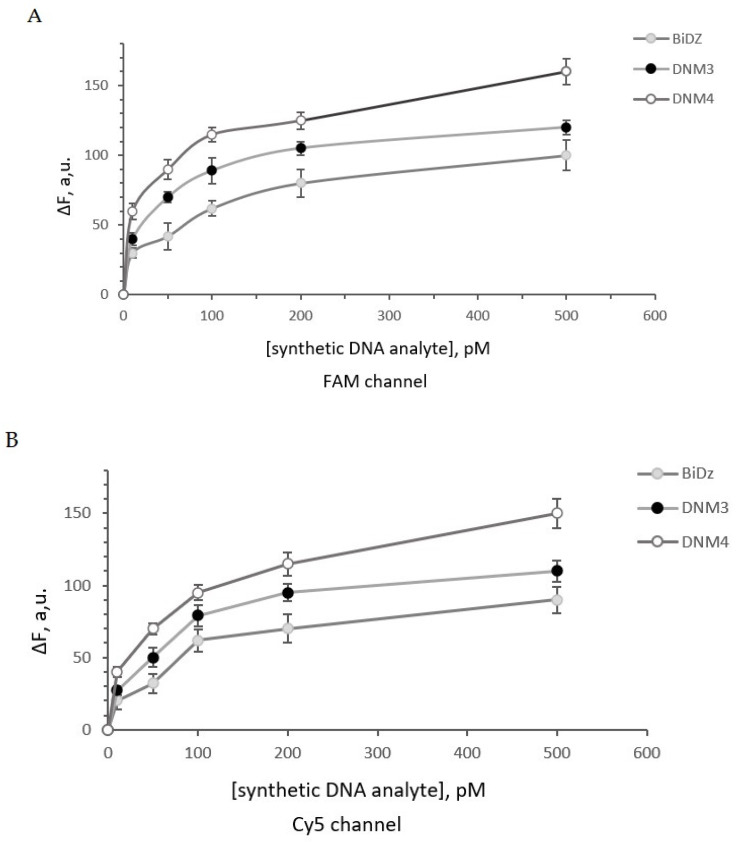
Fluorescence response of the sensors in the presence of different concentrations (0–500 pM) of the synthetic analytes (Analyte-thu and Analyte-myc, Table S1) after 1 h of incubation at 55 °C. (**A**) Response of *B. thuringiensis*–specific DNA nanosensors in the FAM channel. (**B**) Response of *B. mycoides*–specific DNA nanosensors in the Cy5 channel. Average values of three independent measurements are presented with one standard deviation. Tables S2 and S3 contain data correspond to Figure 3A and Figure 3B, respectively.

Initially, the DNA nanosensors were characterized using two 92 nt synthetic DNA oligonucleotides corresponding to 138–237 nt fragment of both *B. thuringiensis* and *B. mycoides* 16S rRNA (Table S1). The *B. thuringiensis* synthetic analyte folded into a stable secondary structure (∆G = −8.77 kcal/mol) (Figure S5A) while the *B. mycoides* synthetic analyte folded into a more stable secondary structure (∆G = −9.35 kcal/mol) (Figure S5B) under the assay conditions. The correct assembly of DNM3 and DNM4 were confirmed by electrophoresis in PAGE gel (Figure 2C). It was demonstrated that all DNA nanosensors responded to the presence of the specific synthetic DNA analyte in a concentration-dependent manner (Figure 3). The limit of detection (LOD) for the DNA nanosensors was found to be in the range of 20–110 pM as shown in Table 2 (Figures S2 and S3).

These data indicate that the LOD for synthetic DNA analyte was only slightly improved by increasing the number of analyte-binding arms. The fluorescence signal at the background level was observed for all the DNA nanosensors targeting *B. thuringiensis* 16S rRNA in the presence of nonspecific *B. mycoides* synthetic analyte and vice versa. This is consistent with the high selectivity of the sensors reported earlier.

### 2.3. Selectivity of the DNA-Nanosensors

The sequence of the targeted 16S rRNAs of the *Bacillus cereus* group species shows a similarity of >99% [54]. In fact, the 16S rRNA sequences of *B. thuringiensis* and *B. mycoides* differ from each other and from the sequences of *B. cereus* and *B. anthracis* by only four to nine nucleotides [54]. Since the mutations are scattered throughout the 16S rRNA sequence, the sensor should have single nucleotide selectivity. Splitting the functional roles between several components of the sensor allows for the fine tuning of the sensor’s characteristics, including selectivity [35]. For example, to make the sensors more selective, it was necessary to shorten the analyte-binding arm of the Dz_a_ strand from 19 to 14 nucleotides (nt) for the sensors targeting *B. thuringiensis* 16S rRNA and from 20 to 15 nt for the sensors targeting *B. mycoides* 16S rRNA, since the sensors with long analyte-binding arms could not reliably discriminate the perfectly complementary analyte from the analyte containing a single mismatch.

**Figure 4:**
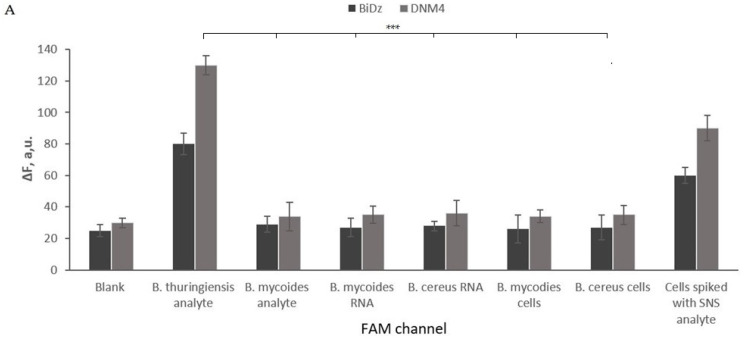

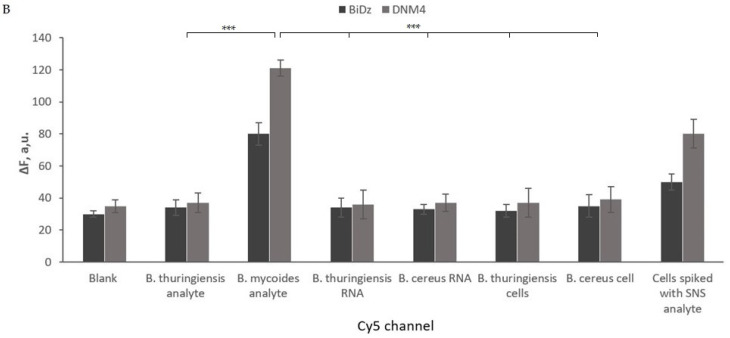
Selectivity of the BiDz and DNM1 sensors. (**A**) Selectivity of *B. thuringiensis*–specific BiDz (dark gray bars) and DNM4 (light gray bars) in the presence of either synthetic oligonucleotides analyte (*B. thuringiensis* or *B. mycoides* analyte) (200 pM), total RNA from either *B. mycoides* or *B. cereus* (2 ng, 1.5–1.6 million of genome equivalents (GE) of *B. cereus* and *B. mycoides*, respectively), *B. mycoides* or *B. cereus* cell and *B. mycoides* cells spiked with *B. thuringiensis* analyte (200 pM after 1 h of incubation at 55 °C in the FAM channel). Blank sample contained no analyte. (**B**) Same as in panel A, but for *B. mycoides* –specific BiDz and DNM4. The samples contained 2 ng (1.4 million GE) of *B. mycoides* total RNA corresponds. The signal was read at Cy5 channel. Average values of three independent measurements are presented with one standard deviation. ***: *p* value < 0.001. Tables S4 and S5 contain data correspond to Figure 4A and Figure 4B, respectively.

The selectivity of the truncated sensors was tested and adjusted using synthetic DNA oligonucleotide analytes mimicking the targeted fragment of 16S rRNAs (positions 138–237). It was shown that for all the sensors, the high fluorescent signal was observed only in the presence of the fully complementary analyte, while the analyte forming a single-base mismatch with the sensor triggered the signal close to the background (Figure 4), even when it was tested at saturation levels. These data are in agreement with the high selectivity of binary probes reported earlier [45]. The DNMs successfully detected the corresponding total RNA extracted from *B. thuringiensis*, *B. mycoides*, and *B. cereus* cultures. *B. thuringiensis*–specific DNM4 emits a signal only in the presence of *B. thuringiensis* RNA, and *B. mycoides*–specific DNM4 in the presence of *B. mycoides* RNA. Neither *B. thuringiensis*–specific nor *B. mycoides*–specific DNM4 detected the *B. cereus* RNA (Figure 4).

### 2.4. Detection of the Bacterial 16S rRNAs Using the DNA-Nanosensors

The performance of *B. thuringiensis*– and *B. mycoides*–specific DNA nanosensors was evaluated in the presence of 10 ng of total RNA isolated from *B. mycoides*, *B. thuringiensis*, or *B. cereus* (Figure S3). It was found that the BiDz sensors showed a signal as low as the background with the three different totals of RNA even after 3 h of incubation.

**Figure 5:**
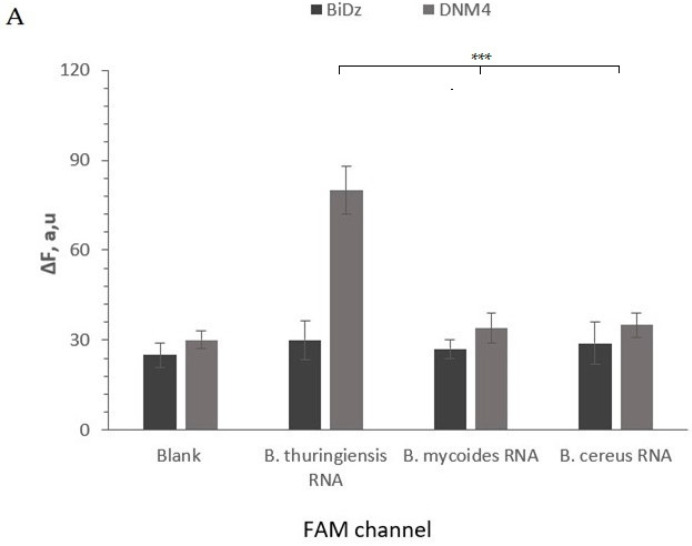

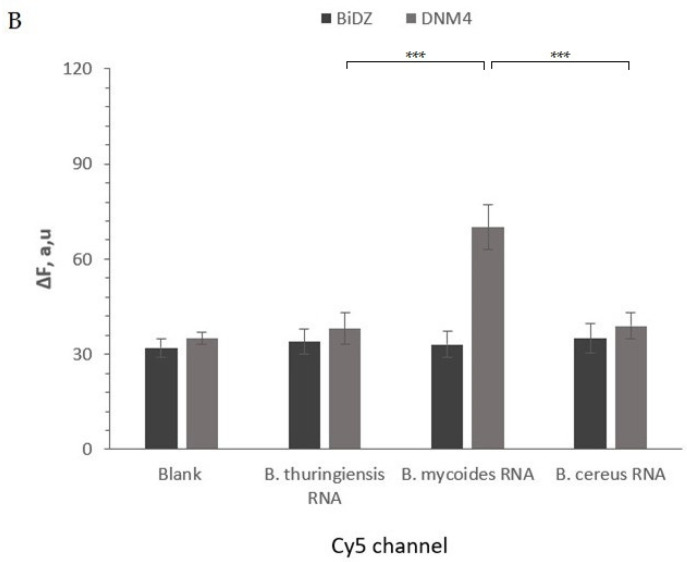
Analysis of the total bacterial RNA using BiDz and DNM4. (**A**) Fluorescence intensities of *B. thuringiensis*–specific BiDz (dark gray bars) and DNM4 (light gray bars) in the absence or the presence of 2 ng of *B. thuringiensis* RNA, *B. mycoides* RNA, and *B. cereus* RNA after 1 h of incubation at 55 °C in the FAM channel. (**B**) Fluorescence intensities of *B. mycoides*–specific BiDz (dark gray bars) and DNM4 (light gray bars) in the absence or the presence of 2 ng of *B. thuringiensis* RNA, *B. mycoides* RNA, and *B. cereus* RNA after 1 h of incubation at 55 °C in the Cy5 channel. Average values of three independent measurements are presented with one standard deviation. ***: *p* value < 0.001. Tables S6 and S7 contain data correspond to Figure 5A and Figure 5B, respectively.

A 1.5-fold increase in the fluorescence was observed for the DNM3 but only after 3 h of incubation (Figures S6 and S7), whereas the DNM4 showed a 1.5-fold increase in the fluorescence in the presence of the target RNA after 1 h of incubation. The *B. mycoides*– and *B. thuringiensis*–specific DNA nanosensors showed fluorescence at the background level in the presence of non-target RNA (*B. cereus*) (Figure 5). The LOD of the DNM4 was calculated since it showed the best performance out of the three variants. It was found to be in the range of 1–1.5 ng, which corresponds to ~5 × 10^8^ of 16S rRNA molecules of the target 16S rRNA after 1 h of incubation (Figure S8).

### 2.5. Detection of the 16S rRNAs Using the B. thuringiensis and B. mycoides in Crude Cell Lysates

**Figure 6:**
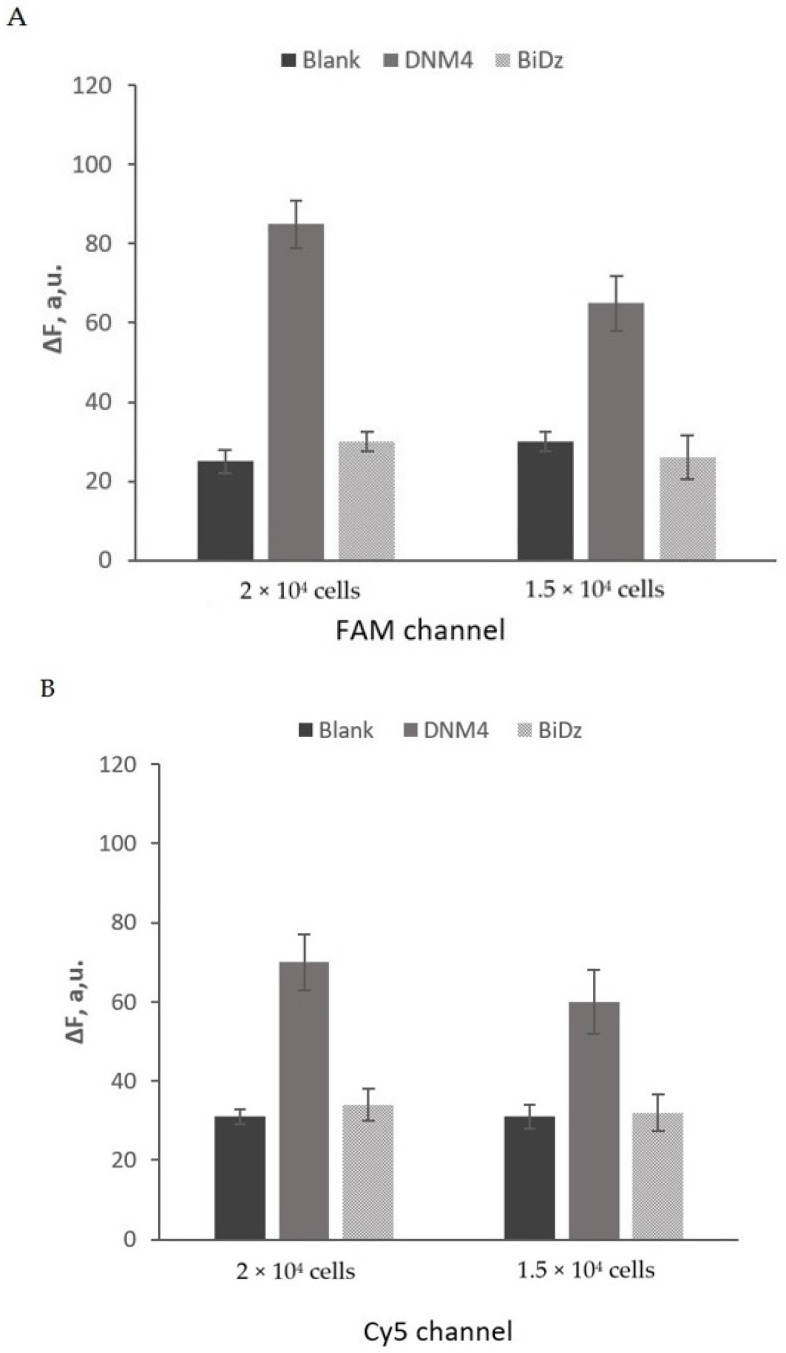
Response of DNM4 and BiDz to cell lysate. (**A**) Fluorescent intensity of Blank (no analyte) (dark gray bars), *B. thuringiensis*–specific DNM4 (light gray bars), or BiDz (striped bars) in the presence of *B. thuringiensis* cells (2 × 10^4^ or 1.5 × 10^4^ cells per reaction volume) in the FAM channel after 1 h of incubation at 55 °C. (**B**) Fluorescent intensity of Blank (dark gray bars), *B. mycoides*–specific DNM4 (light gray bars), or BiDz (light grey bars) in the presence of *B. mycoides* cells (2 × 10^4^ or 1.5 × 10^4^ cells per reaction volume) in the Cy5 channel after 1 h of incubation at 55 °C. Average values of three independent measurements are presented with one standard deviation. Tables S8 and S9 contain data correspond to Figure 6A and Figure 6B, respectively.

Relying on the high selectivity of the DNM4 sensor, we used it with crude cell lysates without isolating target RNAs. We tested serial dilutions of a *B. thuringiensis* and *B. mycoides* cell culture (OD600 of 1–1.2) to demonstrate the performance of the DNM4 sensor in comparison with the BiDz sensor (Figure 6). The sensors detected 2 × 10^4^ cells with S/B of ~3, which is good enough for distinguishing a true signal from the noise in fluorescent assays as was discussed earlier [43].

The fluorescence response of the sensor’s DNM4 increased with increased cell numbers, reaching saturation when 8 × 10^8^ cells per reaction volume or more were used. At the same time, the BiDz sensor showed a response as low as the background (Figure 6, light grey bars). The response of *B. thuringiensis*–specific DNM4 to nonspecific *B. mycoides* and *B. cereus* cells were close to the background (Figure 4A), which also verifies the excellent selectivity of DNM. The response of *B. mycoides*–specific DNM4 to nonspecific *B. thuringiensis* and *B. cereus* cells was close to the background (Figure 4B). The LOD of the DNM4 sensor was found to be 30 × 10^3^ CFU/mL for *B. thuringiensis* and 35 × 10^3^ CFU/mL for *B. mycoides* (Figures S9 and S10).

**Figure 7:**
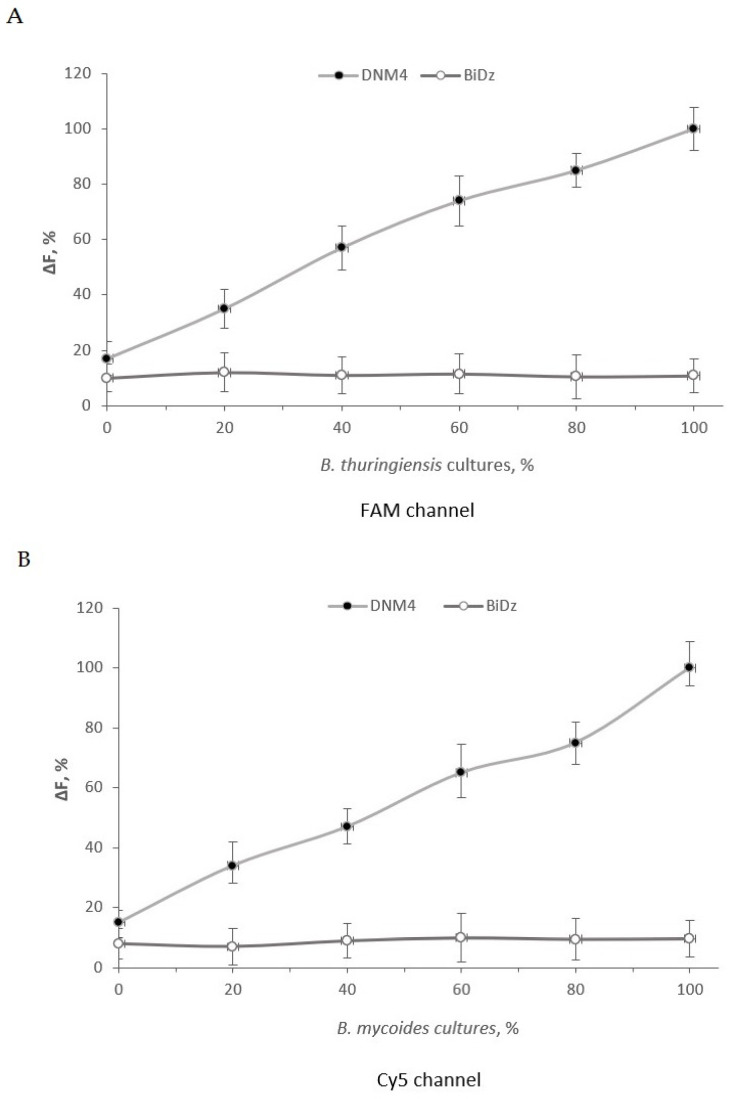
Unlike BiDz, DNM4 can detect specific bacteria in a mixture of cell lysates. (**A**) Normalized fluorescence intensity of either *B. thuringiensis*–specific BiDz (open circles) or DNM4 (filled circles) sensor in the presence of mixtures of *B. thuringiensis* cells containing a different amount of *B. mycoides* and *B. cereus* cells in the FAM channel after 1 h of incubation at 55 °C. The fluorescence values were normalized by either the averaged fluorescence for all samples (for BiDz) or maximum fluorescence (for DNM4) achieved in the presence of 100% of *B. thuringiensis* cells (3 × 10^5^ CFU). (**B**) Normalized fluorescence intensity of either *B. mycoides*–specific BiDz (open circles) or DNM4 (filled circles) sensor in the presence of mixtures of *B. mycoides* cells containing a different amount of *B. thuringiensis* and *B. cereus* cells in the Cy5 channel after 1 h of incubation at 55 °C. The fluorescence values were normalized by either the averaged fluorescence for all samples (for BiDz) or maximum fluorescence (for DNM4) achieved in the presence of 100% of *B. mycoides* cells (3 × 10^5^ CFU). Average values of three independent measurements are presented with one standard deviation.

To demonstrate the applicability of the DNM sensors in cell culture with a mixture of *B. thuringiensis*, *B. mycoides*, and *B. cereus* rRNA, we prepared samples of *B. thuringiensis* cells containing different fractions of *B. mycoides* and *B. cereus* cells and samples of *B. mycoides* cells containing different fractions of *B. thuringiensis* cells and *B. cereus* cells. The samples were analyzed using the DNM4 and BiDz sensors. The fluorescent response of BiDzs was close to the background. As expected, the fluorescence intensity of DNMs increased with increasing percentage of the bacterial cells with maximum fluorescence triggered by the sample containing 100% of the cells (Figure 7).

## 3. Discussion

BiDz technology was introduced in 2007 as a protein-free signal amplification technique with a potential for amplification-free detection of nucleic acids [42]. The major advantage of this technology was the unprecedented high selectivity of SNV detection due to the split design [45]. Since then, achieving the lowest possible LOD for BiDz has been an important goal in developing the technology [53]. With the discovery of 10–23 BiDz by Mokany et at. [43], the technology received attention due to achieving an impressive 5 pM LOD after 3 h of incubation at 55 °C with short synthetic DNA analytes. This LOD was reduced to 2 pM within a 2 h timeframe by the Dz cascade for signal amplification [55]. BiDz equipped with a DNA antenna tile for F-sub delivery enabled a LOD of ~5 pM after only 1 h of incubation [56]. BiDz probe has been demonstrated to be efficient in detecting short DNA analytes [57,58,59], but the performance is less in case of long biological RNA. Being the most sensitive probe among protein enzyme-free approaches, BiDz is not sensitive enough to detect a majority of biological DNA or RNA analytes without amplification, including the mRNA responsible for bacteria drug resistance. Indeed, while the PCR-based method can detect down to 1–10 analytes due to exponential amplification, DNM4 detected only ~5 × 10^8^ of 16S rRNA molecules (Figure S8) in this study due to just linear signal amplification. One important exception for this limitation is the detection of 16S rRNA, which is present in thousands of copies in a single bacteria cell. We believe that developing an amplification-free technique for detecting bacteria based on 16S rRNA-specific BiDz is feasible.

However, analysis of such a long biologically folded RNA as 16S rRNA by traditional hybridization probes has always been a major challenge [60]. The main problem is that the targeted fragments are involved in an intramolecular base paring and, therefore, they are hardly accessible. Secondary structure in analytes has been found to significantly reduce probe-target duplex formation and slow hybridization kinetics [61,62,63]. Amplification of such RNA samples requires a PCR instrument and specialized diagnostic laboratory, which makes the analysis procedure costly and time consuming. To enable amplification-free detection of 16S rRNA, we equipped BiDz with additional RNA-binding arms. This modification resulted in a thermodynamically favorable RNA/DNM complex, which led to the unfolding of the RNA secondary structure, thus reducing the time of the assay and achieving the lowest possible LOD. The strategy reported here can be further adapted from biplex to multiplex system targeting more than two analytes, since the adaptation would require only a new fluorogenic substrate and modification of the substrate-binding arms.

The use of the 16S rRNA sequence as a reliable marker for the discrimination of *B. cereus* group members has failed in previous studies [54,64] due to the high nucleotide conservation in the sequence among the species of this group. Detection and differentiation of *B. cereus* group species in routine diagnostics can be difficult and time- and reagent-consuming since current species’ designations are linked to the specific phenotypic characteristic or the presence of species-specific genes. To the best of our knowledge, this is the first attempt to differentiate *B. cereus* species with an amplification-free approach. The DNM approach uses inexpensive reagents and equipment; it does not require perishable reagents (e.g., protein enzymes) and provides impressive selectivity towards SNV. Therefore, DNM technology promises to be less expensive and more deliverable to point-of-care settings than current gold diagnostic standard qPCR and new generation sequencing (NGS).

DNM4 detected ~1.5 ng of total RNA (Figure S6), which corresponds to ~5×10^8^ 16S rRNA molecules. The same sensor detected ~30,000 colony-forming units in crude cell extract (Figures S9 and S10). Wei el al. [65] reported a RT-qPCR-based method for the detection of *B. thuringiensis* with an LOD that can reach down to 10^3^ CFU/g of *B. thuringiensis* in spiked food samples. There is no well-established infectious dose of *B. thuringiensis* or *B. cereus* partly due to the large differences in the amount of enterotoxin produced by different strains [66]. However, counts ranging from 200 to 10^9^ g^−1^ (or mL^−1^) *B. cereus* have been reported in affected food after food poisoning. Thus, the total infective dose seems to vary between 10^5^–10^8^ viable cells or spores [67]. Despite this, further improvement in DNM sensitivity is needed. It can include using multiple DNMs for binding the same RNA molecule or adapting the antenna tile design for facilitated substrate delivery to the activated Dz core [56] or adding more analyte-binding arms. The DNMs proposed here can become an advantageous tool for detecting SNV in clinically significant DNA or RNA samples and can easily differentiate SNV under broadly variable experimental conditions [68,69].

## 4. Materials and Methods

### 4.1. Materials

The fluorescence susbstrates were purchased in DNA-synthesis, Moscow, Russia. Other oligonucleotides were purchased in Evrogen, Moscow, Russia. DNAse/RNAse free water was purchased from Qiagen, Hilden, Germany. Salts, including MgCl_2_, NaCl, KCl, HEPES, and DEPC were purchased from CarlROTH, Carlsruhe, Germany. Acrylamide was purchased from Applichem, Illinois, USA. Other materials for electrophoresis, including agarose, tris, boric acid, EDTA, bisacrylamide, APS, TEMED, ethidium bromide, and SDS were purchased from Helicon, Moscow, Russia. Ladders and gel loading dyes were purchased from Evrogen, Moscow, Russia. Consumables for bacteria growth, including agar, tripton and yeast extract, were purchased in Dia-M, Moscow, Russia. Glass beads of technical grade was a gift from a technical department. Sodium acetate, phenol, and chlorophorm were purchased from Lenreactiv, Saint-Petersburg, Russia.

### 4.2. Assembling of DNMs

Stock solutions of DNM1_thu, DNM1-1_thu, DNM1_myc, and DNM1-1_myc were prepared by annealing 10 nM of (T1_thu and T2_thu), (T1_thu and T2′_thu), (T1_myc and T2_myc), and (T1_myc and T2′_myc) in the reaction buffer (200 mM MgCl_2_, 150 mM KCl, 15 mM NaCl, and 50 mM HEPES, pH 7.4). For all the experiments, we used 20 nM of DNM1_thu and DNM1-1_thu containing 20 nM of Dza_thu strand and 15 nM of DNM1_myc and DNM1-1_myc containing 20 nM of Dza_myc strand. These concentrations of DNMs and DZas were found to be optimal in terms of the analyte-specific dependent response over the background.

### 4.3. General Fluorescence Assay for Measuring the Limit of Detection (LOD)

Each sample was prepared in 150 µL of reaction buffer (200 mM MgCl_2_, 150 mM KCl, 15 mM NaCl, and 50 mM HEPES, pH 7.4) containing 200 nM F-sub or Cy-sub, 20 nM DNM_thu and 20 nM DZa_thu or 15 nM DNM_myc and 20 nM DZa_myc, and specific synthetic analyte in concentrations ranged from 0 pM to 800 pM. When the assay was performed with either *B. thuringiensis*, *B. mycoides*, or *B. cereus* total RNA, the concentration ranged from 0.1 ng to 20 ng. When the assay was performed with cell lysates, the cells were first pelleted by centrifugation and resuspended in the reaction buffer containing DNM and DZa. The samples were heated for 5 min at 95 °C to enable cell lysis and centrifuged to pellet the cell debris, and the supernatant was transferred to a clean tube before adding the reporter to minimize the background. All samples were incubated at 55 °C for 60 or 180 min followed by fluorescent measurement. This measurement was performed at Spark, Tecan, Switzerland fluorimeter at wavelengths (ex/em) 480 nm/525 nm for F-sub and 617/662 nm for Cy-sub.

### 4.4. Preparation and Analysis of Total RNA

Total RNA was isolated from *Bacillus cereus* ATCC 14579, *Bacillus thuringiensis* ATCC 10792, and *Bacillus mycoides* ATCC 6462 according to the method developed by Oh and So [70] (Figure S1). Namely, the cells of *B. thuringiensis* and *B. cereus* were grown in 3 mL LB at 37 °C for 16 h while the cells of *B. mycodies* were grown in 3 mL LB at 30 °C. Then the cells were harvested by centrifugation at 10,000× *g* for 3 min. The cell pellets were resuspended in a lysis solution of 800 µL (sodium acetate 2.7 g, SDS 5 g, EDTA 0.34 g per liter of deionized water, pH 5.5). Unless otherwise specified, all procedures were conducted at room temperature. The acid pre-treated glass beads (0.8 g, 425–600 um in diameter) were added in the cell suspensions and the samples were vortexed for 2–3 min at 2000 rpm to break the cell wall. The samples were centrifuged at 10,000× *g* for 3 min and the supernatants (500 µL) were transferred to fresh 1.5 mL microtubes. The supernatants were mixed with 500 µL of saturated phenol (pH 5.5) and incubated at 68 °C for 5 min with periodic mixing. Samples were then centrifuged at 10,000× *g* for 3 min. The aqueous layer (450 µL) was transferred to a 1.5 mL fresh microtube and mixed with chloroform (450 µL) before being centrifuged at 10,000× *g* for 3 min. The aqueous layer (400 µL) was transferred to a 1.5 mL fresh microtube and mixed with 100% ethanol (800 µL) and 3 M sodium acetate (1/10 volume). Then RNA pellets obtained by centrifugation at 10,000× *g* for 10 min, were washed with ice-cold 70% ethanol, and dried in a speed vacuum. Dried RNA samples were resuspended in 45 µL of nuclease-free water. The samples were visualized by electrophoresis in 1% agarose at 100 V during 30 min followed by staining in Ethidium Bromide Stain for 15 min.

### 4.5. Preparation and Analysis of the Whole Bacterial Cells

*B. thuringiensis* was inoculated with 200 mL of Lysogeny broth (LB broth) and cultivated overnight at 37 °C. The cells were diluted to reach OD600 of ~1.1. To perform the calibration up to higher OD, 1 mL of the culture was centrifuged, the supernatant was removed, and another 1 mL culture was added. The sample was centrifuged again, the supernatant was removed, and 1 mL saline was added, and the cells were re-suspended. This *B. thuringiensis* stock solution reached an OD600 of 2.2 AU. Serial dilutions were made from the stock solution. The serial dilutions were further diluted by a factor of 80,000 (4 times 10×, followed by a final 8× dilution). A total of 50 µL of the diluted *B. thuringiensis* serial dilutions were plated onto Petri dishes and were incubated overnight at 37 °C and the colony forming units were counted. Only plates between 30 and 300 CFU were used for the calculation of the cell numbers. The cell number as CFU/mL was calculated by multiplication of the counted CFU on the Petri dish with the dilution factor of 80,000 and multiplication of 20 to correct for the CFU per mL. The same procedure was repeated to calculate the number of *B. mycoides* cells.

For the assay, the serial dilutions were prepared in 1 mL per sample and the cells were collected by centrifugation at 900× *g* for 5 min. The supernatant was discarded, and the cell pellet was resuspended in 150 µL of the reaction buffer containing either *B. thuringiensis*–specific or *B. mycoides*–specific DNM1 and BiDz. To ensure the cell lysis and liberation of the RNA from the cells and to anneal it with the deoxyribozyme strands, the mixture was heated at 95 °C for 5 min, left to cool down at room temperature for 5 min, and then centrifuged at 900× *g* for 3 min. The supernatant (~140 µL) was transferred into a clean tube before adding 1 µL of RNase inhibitor and the reporter to minimize the background. All samples were incubated at 55 °C for 60 or 180 min followed by fluorescent measurement.

### 4.6. Preperation and Analysis of Cell Mixtures

To prepare the samples of cell mixtures, *B. thuringiensis* cells, *B. mycoides* cells, and *B. cereus* cells were grown separately to OD600 of ~1 and then all cell cultures were diluted 50-fold with medium. Two series of diluted cultures were prepared. The first series of diluted cultures were mixed in 0:0:10, 1:1:8, 2:2:6, 3:3:4, 4:4:2, or 5:5:0 ratio to obtain samples of *B. thuringiensis* cultures containing 100%, 80%, 60%, 40%, 20%, or 0% of the *B. thuringiensis* cells, respectively. The second series of diluted cultures were mixed in 0:0:10, 1:1:8, 2:2:6, 3:3:4, 4:4:2, or 5:5:0 ratio to obtain samples of *B. mycoides* cultures containing 100%, 80%, 60%, 40%, 20%, or 0% of the *B. mycoides* cells, respectively. The samples were analyzed using the DNMs and BiDzs.

### 4.7. Statistical Analysis

A t-test was used to compare the differences between the *B. cereus*, *B. mycoides,* and *B. thuringiensis*. An ANOVA of variance followed by post hoc tests (Bonferroni) was used to compare differences between different groups. All values are presented as mean ± SD. A *p*-value < 0.05 was considered statistically significant.

## 5. Conclusions

DNA machines for fluorescence analysis of biologically folded RNA were designed and tested. The multi-armed DNM approach enabled the amplification-free detection and discrimination of *B. thuringiensis* and *B. mycoides*. The multi-armed approach does not require long analyte-binding fragments and so allows for the detection of targets with complex secondary and SNVs at the same time. The assay enabled expedite quantification of rRNA content in cell cultures starting from whole cells with LOD down to 30,000 bacterial cells. The method requires 1.5 h with a hands-on time of ~10 min, which outperforms other commercially available tests. The approach is sensitive to SNVs. Further development of the technology should lower the LOD and shorten the assay time to 15–30 min, which could make it competitive with state-of-the-art detection techniques. The same approach is not limited to the *Bacillus cereus* species and can be later expanded to apply to other difficult-to-discriminate targets.

## Figures and Tables

**Table 1:** Comparison between the 16S rRNA SNV-containing fragments of *B. thuringiensis, B. mycoides*, and *B. cereus.*

Species	Sequence
*Bacillus thuringiensis*	A**C**AUUUUGAACUGCAU**G**G
*Bacillus mycoides*	A**U**AUUUUGAAC**U**GCAU**A**G
*Bacillus cereus*	A**C**AUUUUGAAC**C**GCAUGG

SNV 192 that was subject to differentiation is shown in bold blue color. SNV 197 is shown in bold red color. SNV 182 is shown in bold green color [54].

**Table 2:** Limit of detection for all the DNA nanosensors for the synthetic analyte.

Sensor	Analyte	LOD, pM	
		Incubation Time	
		60 min	180 min
BiDz	*B. thuringiensis* DNA	100	55
	*B. mycoides* DNA	110	80
DNM3	*B. thuringiensis* DNA	65	35
	*B. mycoides* DNA	75	50
DNM4	*B. thuringiensis* DNA	40	20
	*B. mycoides* DNA	50	25

## Data Availability

Not applicable.

## Supplementary Materials

The following supporting information can be downloaded at: https://www.mdpi.com/article/10.3390/ijms24054473/s1. References [71,72] are cited in the Supplementary Materials.

## References

[1] Tewari A., Abdullah S. (2015). Bacillus cereus food poisoning: International and Indian perspective. J. Food Sci. Technol..

[2] Holmes J.R., Plunkett T., Pate P., Roper W.L., Alexander W.J. (1981). Emetic food poisoning caused by *Bacillus cereus*. Arch. Intern. Med..

[3] Fritze D. (2004). Taxonomy of the genus bacillus and related genera: The aerobic endospore-forming bacteria. Phytopathology.

[4] Jessberger N., Dietrich R., Granum P.E., Märtlbauer E. (2020). The Bacillus cereus Food Infection as Multifactorial Process. Toxins.

[5] Bravo A., Likitvivatanavong S., Gill S.S., Soberón M. (2011). Bacillus thuringiensis: A story of a successful bioinsecticide. Insect Biochem. Mol. Biol..

[6] Schnepf E., Crickmore N., Van Rie J., Lereclus D., Baum J., Feitelson J., Zeigler D.R., Dean D.H. (1998). Bacillus thuringiensis and its pesticidal crystal proteins. Microbiol. Mol. Biol. Rev..

[7] Melo A.L., Soccol V.T., Soccol C.R. (2016). Bacillus thuringiensis: Mechanism of action, resistance, and new applications: A review. Crit. Rev. Biotechnol..

[8] Johler S., Kalbhenn E.M., Heini N., Brodmann P., Gautsch S., Bağcioğlu M., Contzen M., Stephan R., Ehling-Schulz M. (2018). Enterotoxin Production of Bacillus thuringiensis Isolates From Biopesticides, Foods, and Outbreaks. Front. Microbiol..

[9] Liu Y., Lai Q., Göker M., Meier-Kolthoff J.P., Wang M., Sun Y., Wang L., Shao Z. (2015). Genomic insights into the taxonomic status of the Bacillus cereus group. Sci. Rep..

[10] Ehling-Schulz M., Lereclus D., Koehler T.M. (2019). The *Bacillus cereus* Group: Bacillus Species with Pathogenic Potential. Microbiol. Spectr..

[11] Bourque S.N., Valero J.R., Lavoie M.C., Levesque R.C. (1995). Comparative analysis of the 16S to 23S ribosomal intergenic spacer sequences of Bacillus thuringiensis strains and subspecies and of closely related species. Appl. Environ. Microbiol..

[12] Frederiksen K., Rosenquist H., Jørgensen K., Wilcks A. (2006). Occurrence of natural Bacillus thuringiensis contaminants and residues of Bacillus thuringiensis-based insecticides on fresh fruits and vegetables. Appl. Environ. Microbiol..

[13] Reyes-Ramírez A., Ibarra J.E. (2008). Plasmid patterns of Bacillus thuringiensis type strains. Appl. Environ. Microbiol..

[14] Zheng J., Peng D., Ruan L., Sun M. (2013). Evolution and dynamics of megaplasmids with genome sizes larger than 100 kb in the Bacillus cereus group. BMC Evol. Biol..

[15] Porcar M., Juárez-Pérez V. (2003). PCR-based identification of Bacillus thuringiensis pesticidal crystal genes. FEMS Microbiol. Rev..

[16] Francisco Castillo-Esparza J., Luévano-Borroel J., Ibarra J.E. (2021). Identification and characterization of a new cry-like gene found in a Bacillus cereus strain. Antonie Van Leeuwenhoek.

[17] Barloy F., Delécluse A., Nicolas L., Lecadet M.M. (1996). Cloning and expression of the first anaerobic toxin gene from *Clostridium bifermentans* subsp. malaysia, encoding a new mosquitocidal protein with homologies to Bacillus thuringiensis delta-endotoxins. J. Bacteriol..

[18] Yokoyama T., Tanaka M., Hasegawa M. (2004). Novel cry gene from *Paenibacillus lentimorbus* strain *Semadara inhibits* ingestion and promotes insecticidal activity in Anomala cuprea larvae. J. Invertebr. Pathol..

[19] Böhm M.E., Huptas C., Krey V.M., Scherer S. (2015). Massive horizontal gene transfer, strictly vertical inheritance and ancient duplications differentially shape the evolution of *Bacillus cereus* enterotoxin operons hbl, cytK and nhe. BMC Evol.Biol..

[20] Osman G., Already R., Assaeedi A., Organji S., El-Ghareeb D., Abulreesh H., Althubiani A.S. (2015). Bioinsecticide *Bacillus thuringiensis* a comprehensive review. Egypt. J. Biol. Pest Control.

[21] Cho S.H., Kang S.H., Lee Y.E., Kim S.J., Yoo Y.B., Bak Y.S., Kim J.B. (2015). Distribution of Toxin Genes and Enterotoxins in *Bacillus thuringiensis* Isolated from Microbial Insecticide Products. J. Microbiol. Biotechnol..

[22] Pfrunder S., Grossmann J., Hunziker P., Brunisholz R., Gekenidis M.T., Drissner D. (2016). *Bacillus cereus* Group-Type Strain-Specific Diagnostic Peptides. J. Proteome Res..

[23] Busch A., Schotte U., Jeßberger N., Frentzel H., Plötz M., Abdulmawjood A. (2022). Establishment and validation of a two-step LAMP assay for detection of *Bacillus cereus*-group isolates in food and their possibility of non-haemolytic enterotoxin production. Front. Microbiol..

[24] Zheng H., Sheng R., Li H., Chen Q. (2022). Rapid and selective detection of Bacillus cereus in food using cDNA-based up-conversion fluorescence spectrum copy and aptamer modified magnetic separation. Spectrochim. Acta Part A Mol. Biomol. Spectrosc..

[25] Moteshareie H., Hassen W.M., Dirieh Y., Groulx E., Dubowski J.J., Tayabali A.F. (2022). Rapid, Sensitive, and Selective Quantification of *Bacillus cereus* Spores Using xMAP Technology. Microorganisms.

[26] Trinh T.N.D., Lee N.Y. (2021). Nucleic acid amplification-based microfluidic approaches for antimicrobial susceptibility testing. Analyst.

[27] Zheng C., Wang K., Zheng W., Cheng Y., Li T., Cao B., Jin Q., Cui D. (2021). Rapid developments in lateral flow immunoassay for nucleic acid detection. Analyst.

[28] Wu Q., Suo C., Brown T., Wang T., Teichmann S.A., Bassett A.R. (2021). INSIGHT: A population-scale COVID-19 testing strategy combining point-of-care diagnosis with centralized high-throughput sequencing. Sci. Adv..

[29] Kang T., Lu J., Yu T., Long Y., Liu G. (2022). Advances in nucleic acid amplification techniques (NAATs): COVID-19 point-of-care diagnostics as an example. Biosens. Bioelectron..

[30] Li J., Macdonald J. (2015). Advances in isothermal amplification: Novel strategies inspired by biological processes. Biosens. Bioelectron..

[31] Veselinyová D., Mašlanková J., Kalinová K., Mičková H., Mareková M., Rabajdová M. (2021). Selected In Situ Hybridization Methods: Principles and Application. Molecules.

[32] Buh Gasparic M., Tengs T., La Paz J.L., Holst-Jensen A., Pla M., Esteve T., Zel J., Gruden K. (2010). Comparison of nine different real-time PCR chemistries for qualitative and quantitative applications in GMO detection. Anal. Bioanal. Chem..

[33] Kolpashchikov D.M. (2019). Evolution of Hybridization Probes to DNA Machines and Robots. Acc. Chem. Res..

[34] Nguyen C., Grimes J., Gerasimova Y.V., Kolpashchikov D.M. (2011). Molecular-beacon-based tricomponent probe for SNP analysis in folded nucleic acids. Chemistry.

[35] Sun S.C., Dou H.Y., Chuang M.C., Kolpashchikov D.M. (2019). Multi-labeled electrochemical sensor for cost-efficient detection of single nucleotide substitutions in folded nucleic acids. Sens. Actuators B Chem..

[36] Herschlag D., Bonilla S., Bisaria N. (2018). The Story of RNA Folding, as Told in Epochs. Cold Spring Harb. Perspect. Biol..

[37] Fallmann J., Will S., Engelhardt J., Grüning B., Backofen R., Stadler P.F. (2017). Recent advances in RNA folding. J. Biotechnol..

[38] Lahoud G., Timoshchuk V., Lebedev A., de Vega M., Salas M., Arar K., Hou Y.M., Gamper H. (2008). Enzymatic synthesis of structure-free DNA with pseudo-complementary properties. Nucleic Acids Res..

[39] Sau S.P., Kumar T.S., Hrdlicka P.J. (2010). Invader LNA: Efficient targeting of short double stranded DNA. Org. Biomol. Chem..

[40] Gupta A., Mishra A., Puri N. (2017). Peptide nucleic acids: Advanced tools for biomedical applications. J. Biotechnol..

[41] Nedorezova D.D., Dubovichenko M.V., Belyaeva E.P., Grigorieva E.D., Peresadina A.V., Kolpashchikov D.M. (2022). Specificity of oligonucleotide gene therapy (OGT) agents. Theranostics.

[42] Kolpashchikov D.M. (2007). A binary deoxyribozyme for nucleic acid analysis. ChemBioChem.

[43] Mokany E., Bone S.M., Young P.E., Doan T.B., Todd A.V. (2010). MNAzymes, a versatile new class of nucleic acid enzymes that can function as biosensors and molecular switches. J. Am. Chem. Soc..

[44] Tian B., Han Y., Wetterskog E., Donolato M., Hansen M.F., Svedlindh P., Strömberg M. (2018). MicroRNA Detection through DNAzyme-Mediated Disintegration of Magnetic Nanoparticle Assemblies. ACS Sens..

[45] Kolpashchikov D.M. (2010). Binary probes for nucleic acid analysis. Chem. Rev..

[46] Gerasimova Y.V., Kolpashchikov D.M. (2013). Folding 16S RNA in a signal-producing structure for detection of bacteria. Angew. Chem., Int. Ed..

[47] Cox A.J., Bengtson H.N., Rohde K.H., Kolpashchikov D.M. (2016). DNA Nanotechnology for Nucleic Acid Analysis: Multifunctional Molecular DNA Machine for RNA Detection. Chem. Commun..

[48] Gerasimova Y.V., Yakovchuk P., Dedkova L.M., Hecht S.M., Kolpashchikov D.M. (2015). Expedited quantification of mutant ribosomal RNA by binary deoxyribozyme (BiDz) sensors. RNA.

[49] Lyalina T.A., Goncharova E.A., Prokofeva N.Y., Voroshilina E.S., Kolpashchikov D.M. (2019). A DNA minimachine for selective and sensitive detection of DNA. Analyst.

[50] Akhmetova M.M., Rubel M.S., Afanasenko O.S., Kolpashchikov D.M. (2022). Barley haplotyping using biplex deoxyribozyme nanomachine. Sens. Actuators Rep..

[51] El-Deeb A.A., Zablotskaya S.S., Rubel M.S., Nour M.A.Y., Kozlovskaya L.I., Shtro A.A., Komissarov A.B., Kolpashchikov D.M. (2022). Toward a Home Test for COVID-19 Diagnosis: DNA Machine for Amplification-Free SARS-CoV-2 Detection in Clinical Samples. ChemMedChem.

[52] Gorbenko D.A., Shkodenko L.A., Rubel M.S., Slita A.V., Nikitina E.V., Martens E.A., Kolpashchikov D.M. (2022). DNA nanomachine for visual detection of structured RNA and double stranded DNA. Chem. Commun..

[53] Gerasimova Y.V., Cornett E.M., Edwards E., Su X., Rohde K.H., Kolpashchikov D.M. (2013). Deoxyribozyme cascade for visual detection of bacterial RNA. Chembiochem.

[54] Ash C., Farrow J.A., Dorsch M., Stackebrandt E., Collins M.D. (1991). Comparative analysis of *Bacillus anthracis*, *Bacillus cereus*, and related species on the basis of reverse transcriptase sequencing of 16S rRNA. Int. J. Syst. Bacteriol..

[55] Bone S.M., Hasick N.J., Lima N.E., Erskine S.M., Mokany E., Todd A.V. (2014). DNA-only cascade: A universal tool for signal amplification, enhancing the detection of target analytes. Anal. Chem..

[56] Cox A.J., Bengtson H.N., Gerasimova Y.V., Rohde K.H., Kolpashchikov D.M. (2016). DNA Antenna Tile-Associated Deoxyribozyme Sensor with Improved Sensitivity. Chembiochem.

[57] Gerasimova Y.V., Hayson A., Ballantyne J., Kolpashchikov D.M. (2010). A single molecular beacon probe is sufficient for the analysis of multiple nucleic acid sequences. Chembiochem.

[58] Gerasimova Y.V., Kolpashchikov D.M. (2010). Nucleic acid detection using MNAzymes. Chem. Biol..

[59] Hasick N., Lawrence A., Ramadas R., Todd A. (2020). Sensitive Detection of Nucleic Acids Using Subzyme Feedback Cascades. Molecules.

[60] Mahmoud K.K., McNeely D., Elwood C., Koval S.F. (2007). Design and performance of a 16S rRNA-targeted oligonucleotide probe for detection of members of the genus Bdellovibrio by fluorescence in situ hybridization. Appl. Environ. Microbiol..

[61] Sekar M.M., Bloch W., St John P.M. (2005). Comparative study of sequence-dependent hybridization kinetics in solution and on microspheres. Nucleic Acids Res..

[62] Kushon S.A., Jordan J.P., Seifert J.L., Nielsen H., Nielsen P.E., Armitage B.A. (2001). Effect of secondary structure on the thermodynamics and kinetics of PNA hybridization to DNA hairpins. J. Am. Chem. Soc..

[63] Armitage B.A. (2003). The impact of nucleic acid secondary structure on PNA hybridization. Drug Discov. Today.

[64] Chen M.L., Tsen H.Y. (2002). Discrimination of Bacillus cereus and Bacillus thuringiensis with 16S rRNA and gyrB gene-based PCR primers and sequencing of their annealing sites. J. Appl. Microbiol..

[65] Wei S., Chelliah R., Park B.J., Kim S.H., Forghani F., Cho M.S., Park D.S., Jin Y.G., Oh D.H. (2019). Differentiation of Bacillus thuringiensis From Bacilluscereus Group Using a Unique Marker Based on Real-Time PCR. Front. Microbiol..

[66] Granum P.E., Doyle M., Beuchat L., Montville T. (1997). Bacillus cereus. Fundamentals in Food Microbiology.

[67] Granum P.E., Lund T. (1997). Bacillus cereus and its food poisoning toxins. FEMS Microbiol. Lett..

[68] Smith A.L., Kolpashchikov D.M. (2017). Divide and Control: Comparison of Split and Switch Hybridization Sensors. ChemistrySelect.

[69] Reed A.J., Sapia R.J., Dowis C., Solarez S., Gerasimova Y.V. (2020). Interrogation of highly structured RNA with multicomponent deoxyribozyme probes at ambient temperatures. RNA.

[70] Oh E.T., So J.S. (2003). A rapid method for RNA preparation from Gram-positive bacteria. J. Microbiol. Methods.

[71] https://github.com/RNAcentral/r2dt.

[72] MacDougall D., Crummett W.B. (1980). Guidelines for data acquisition and data quality evaluation in environmental chemistry. Anal. Chem..

