# Supplementary material for: DNA Nanomachine (DNM) Biplex Assay for Differentiating Bacillus cereus Species
Source: Int J Mol Sci. 2023 Feb 24;24(5):4473. doi: 10.3390/ijms24054473 (PMC10003685; doi:10.3390/ijms24054473)
Supplement: Supplementary file 1 [file ijms-24-04473-s001.zip › ijms-2221433-supplementary.pdf]

International Journal of *Molecular Sciences*

MDPI

Supplementary Material

DNA nanomachine (DNM) Biplex Assay for Differentiating *Bacillus cereus* Species

Muhannad Ateiah [1], Erik R. Gandalipov [1], Aleksandr A. Rubel [2], Maria S. Rubel [1] and Dmitry M. Kolpashchikov [1,3,4,*]

1 Laboratory of Solution Chemistry of Advanced Materials and Technologies, ITMO University, Lomonosova St. 9, St. Petersburg 191002, Russia
2 Laboratory of Amyloid Biology, St. Petersburg State University, Universitetskaya enb. 7-9, St. Petersburg 199034, Russia
3 Chemistry Department, University of Central Florida, 4000 Central Florida Boulevard, Orlando, FL 32816-2366, USA
4 Burnett School of Biomedical Sciences, University of Central Florida, Orlando, 32816 Florida, USA
* Correspondence: dmitry.kolpashchikov@ucf.edu

Table of content:

1. Material and Method.

2. Oligonucleotides used in this study.

3. Analysis of the extracted total RNA from the bacterial strains in 1% agarose gel

4. Limit of the detection of the B. thuringiensis-specific and B. mycoides- specific DNA nanosensors for the synthetic analyte.

5. Secondary structure model of *B. thuringiensis* 16S rRNA with indicated fragments targeted by DNA nanosensors.

6. Prediction of Secondary structure of *B. thuringiensis* and *B. mycoides* synthetic DNA analyte

7. Comparison between the three DNA-nanosensors

8. Limit of the detection of B. thuringiensis-specific and B. mycoides-specific DNM1 for the total RNA.

9. Limit of the detection of B. thuringiensis-specific and B. mycoides-specific DNM4 for the whole bacterial cells

10. Statistics

1. Material and Methods

DNAse/RNAse-free water was purchased from QIAGEN, Germany and used for all stock solutions of oligonucleotides. MQ water was purified via Millipore RiOs-DI 3 Smart and used for buffers and solutions. Fluorogenic substrates (F-sub and Cy-sub) were syn-thesized and HPLC purified by DNA-synthesis Moscow, Russian Federation. All other oligonucleotides (see Table 1 for sequences) were obtained from DNA-synthesis Moscow, Russian Federation. The oligonucleotides were dissolved in DNAse/RNAse-free water and stored at -20 ^{o}C.

The fluorescence intensities of F-sub reporter were measured at 525 nm (excitation wavelength at 480 nm). The fluorescence intensities of Cy-sub reporter were measured at 662 nm (excitation wavelength at 617 nm). Excitation and emission slits were both 10 nm (spectrophotometer Spark, Tecan).

The collection of bacterial strains (*Bacillus cereus* ATCC 14579, *Bacillus thuringiensis* ATCC 10792, and *Bacillus mycoides* ATCC 6462) was kindly provided by Institute of Children Infection, Russian Federation. Cultivation of the bacterial strains was conducted on LB (lysogeny broth) nutrient media that contains 1% tripton, 0,5% yeast extract, 1% NaCl in 15 ml falcons with 3 mL nutrient media. Cultivation was carried out at a 37 ^{o}C temperature overnight in shaker-incubator Biosan with 250 rpm. Samples of cell cultures were taken for RNA isolation from 16 hour of incubation overnight.

2. Table S1. Sequence of oligonucleotides

Copyright: © 2023 by the authors. Licensee MDPI, Basel, Switzerland. This article is an open access article distributed under the terms and conditions of the Creative Commons Attribution (CC BY) license (https://creativecommons.org/licenses/by/4.0/).

Name	Sequence	Purification
**Reporter (F-sub)**	5′- AAG GTT^{FAM} TCC TCg uCC CTG GGC A-BHQ1	HPLC
**BiDz_Dzb_thu**	5′- TGC CCA GGG A *GGCTAGCT* TCA AAA TGT TAT CC GGT A	SD
**Dza_thu**	5′-TCG AAC **C**AT GCA GT *ACAACGA* GAGGAAACCTT	SD
**T1_thu**	5′- TGC CCA GGG A *GGCTAGCT* TCA AAA TGT TAT CCG GTA /HEG/ ACA CTT AGG ACT GCG AGA CCG ATG GTC AAG TCAC	SD
**T2_thu**	5′- GTA AGT GAC AGC CGA AGC CGC CTT T /HEG/GTG ACT TGA CCA TCG GTC TCG CAG TCC TAA GTG T	SD
**T2′_thu**	5′- GTA AGT GAC AGC CGA AGC CGC CTT T /HEG/ GTG ACT TGA CCA TCG GTC TCG CAG TCC TAA GTG T /HEG/ TTA GCC CTG GTT TCC CGG AGT TAT CCC AG	SD
**Analyte-thu**	5′- TAA GAC TGG GAT AAC TCC GGG AAA CCG GGG CTA ATA CCG GAT AAC ATT TTG AAC TGC AT**G** GTT CGA AAT TGA AAG GCG GCT TCG GCT GTC ACT T	SD

**Reporter (Cy-sub)**	5′- ^{Cy5}CAG CAC AAC Cg uCC CTG GGC A-BHQ-2	HPLC
**BiDz_Dzb_myc**	5′- TGC CCA GGG A *GGCTAGCT* TCA AAA TAT TAT CCG GTA TT	SD
**Dza_myc**	5′- ATT TCG AAC **T**AT GCA GT *ACAACGAG* GTTGTGCTG	SD
**T1_myc**	5′- TGC CCA GGG AGG CTA GCT TCA AAA TAT TAT CCG GTA TT /HEG/ AGT GCA ATG CCA GAC TTA GTA CCG ATC GGA TAA CCG TT	SD
**T2_myc**	5′- AAG TGA CAG CCG AAG CCG CCT TTC /HEG/ AAC GGT TAT CCG ATC GGT ACT AAG TCT GGC ATT GCA CT	SD
**T2′_myc**	5′- AAG TGA CAG CCG AAG CCG CCT TTC /HEG/ AAC GGT TAT CCG ATC GGT ACT AAG TCT GGC ATT GCA CT/HEG/ AGC CCC GGT TTC CCG GAG TTA TCC CAG TCT TA	SD
**Analyte-myc**	5′- TAA GAC TGG GAT AAC TCC GGG AAA CCG GGG CTA ATA CCG GAT AAT ATT TTG AAC TGC AT**A** GTT CGA AAT TGA AAG GCG GCT TCG GCT GTC ACT T	SD

The single-nucleotide variation (SNV) site in both the Dza strands and the synthetic analytes are shown in bold. Nucleotides constituting the catalytic core of 10–23 deoxyribozymes are shown in italics. Nucleotides of the Dz strands that are complementary to the reporter are underlined. Ribonucleotides are in lowercase. (FAM) 6-carboxyfluorescein, (BHQ1) Black Hole quencher 1, (Cy5) Cyanine 5, (BHQ2) Black Hole quencher 2, HEG — hexaethyleneglycol, SD: standard desalting.

3. Analysis of the extracted total RNA from the bacterial strains in 1% agarose gel

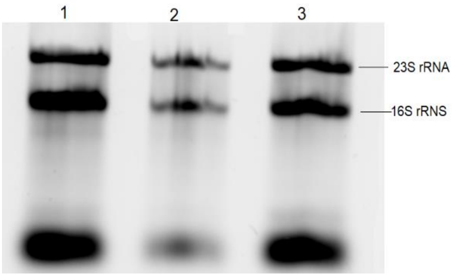

Figure S1. Analysis of the extracted total RNA from the bacterial strains in 1% agarose gel. Lane 1: total RNA extracted from *B. thuringiensis*. Lane 2: total RNA extracted from *B. cereus*. Lane 3: total RNA extracted from *B. mycoides*. The position of 16S rRNA is indicated by an arrow.

4. Limit of the detection of the B. thuringiensis-specific and B. mycoides- specific DNA nanosensors for the synthetic analyte.

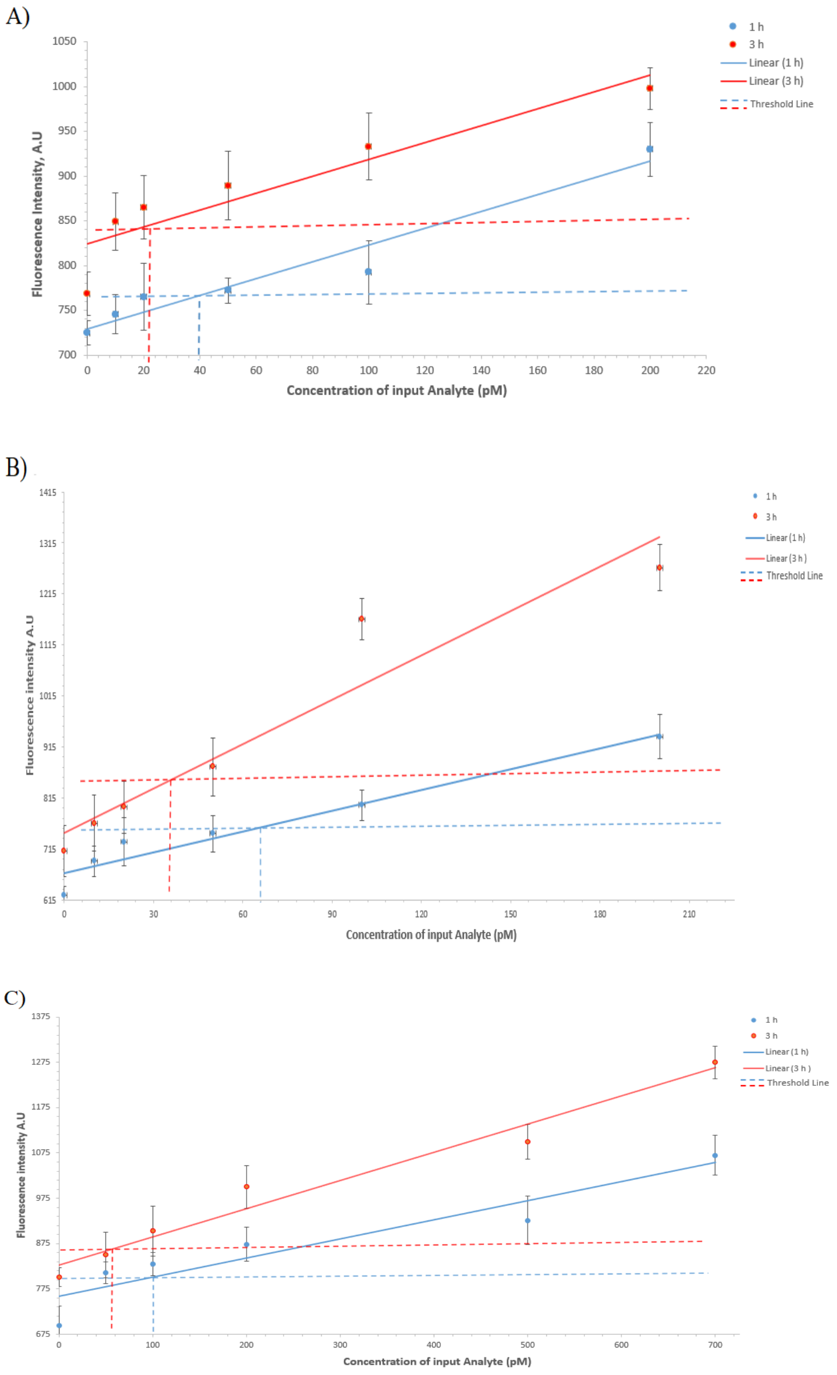

Figure S2. Limit of the detection of *B. thuringiensis*- specific DNA nanosensors for the synthetic analyte. The signal is shown for DNM4 (Figure S2, A), the DNM3 (Figure S2, B) and the BiDz (Figure S2, C). Samples were incubated at 55°C in the reaction buffer (200mM $MgCl_2$, 150 mM KCl, 15 mM NaCl and 50 mM HEPES, pH 7.4) with different concentrations of the analyte (0-700 pM). Fluorescent intensities were measured after 60 min and 180 min. The data are average values of three independent measurements. The errors are given as one standard deviation of the average. The Limit of detection is set as 3 standard deviations above the negative sample.

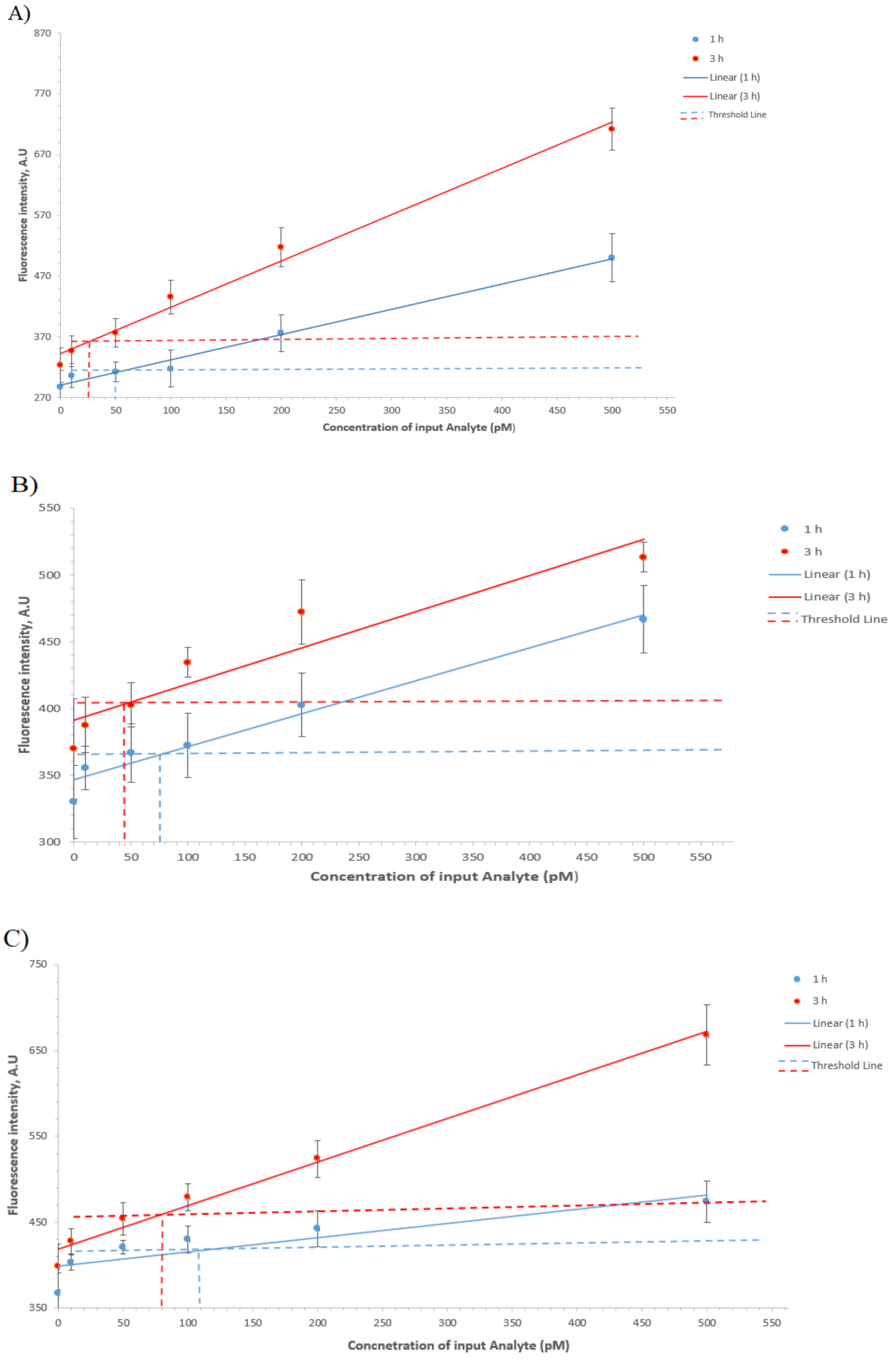

Figure S3. Limit of the detection of *B. mycoides*- specific DNA nanosensors for the synthetic analyte. The signal is shown for DNM4 (Figure S3, A), the DNM3 (Figure S3, B) and the BiDz (Figure S3, C). Samples were incubated at 55°C in the reaction buffer (200 mM $MgCl_2$, 150 mM KCl, 15 mM NaCl and 50 mM HEPES, pH 7.4) with different concentrations of the analyte (0-500 pM). Fluorescent intensities were measured after 60 min and 180 min. The data are average values of three independent measurements. The errors are given as one standard deviation of the average. The Limit of detection is set as 3 standard deviations above the negative sample.

5. Secondary structure model of *B. thuringiensis* 16S rRNA with indicated fragments targeted by DNA nanosensors.

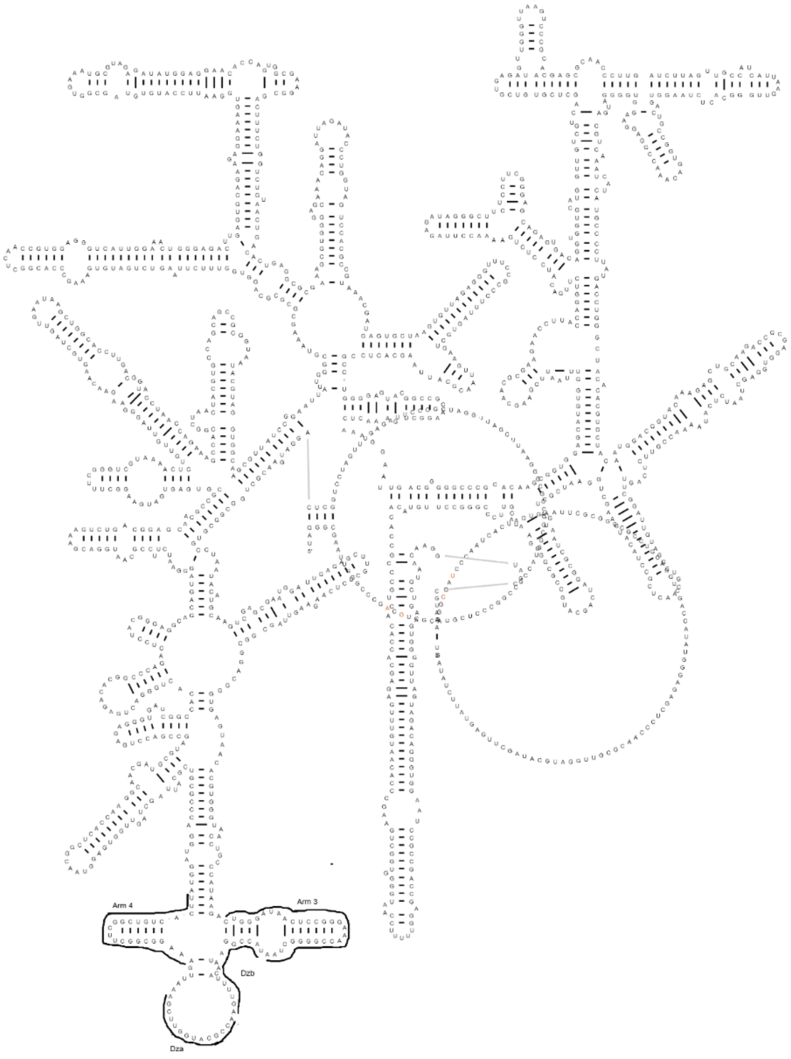

Figure S4. Secondary structure model of *B. thuringiensis* 16S rRNA with indicated fragments targeted by DNA nanosensors. The secondary structure was generated by R2DT using the EC_SSU_3D template provided by RiboVision [71].

6. Prediction of Secondary structure of *B. thuringiensis* and *B. mycoides* synthetic DNA analyte

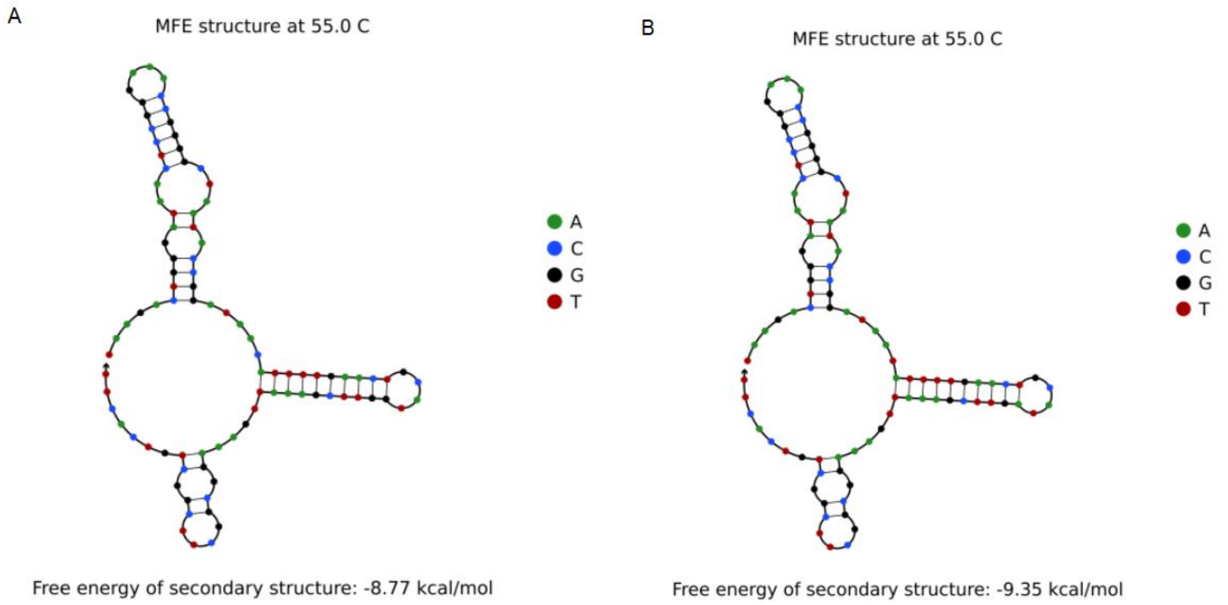

Figure S5. Prediction of Secondary structure of *B. thuringiensis* and *B. mycoides* synthetic DNA analyte. A) Secondary structure of *B. thuringiensis* DNA analyte predicted by Mfold at 55°C in the reaction buffer (100mM Mg ions, 215 mM Na ions). B) Secondary structure of *B. mycoides* DNA analyte predicted by Mfold at 55°C in the reaction buffer (100mM Mg ions, 215 mM Na ions).

7. Comparison between the three DNA-nanosensors

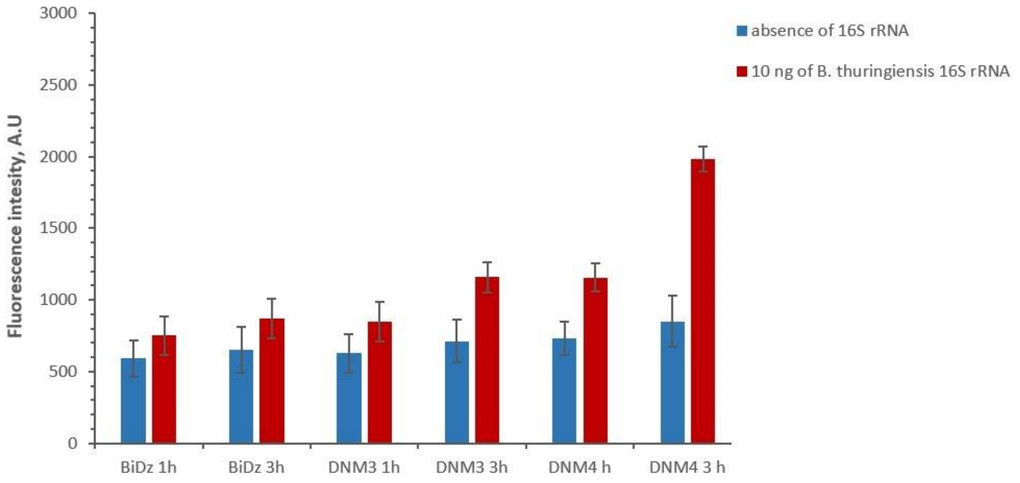

Figure S6. Comparison between the three DNA-nanosensors in detecting the bacterial *B. thuringiensis* 16S rRNA. Fluorescence intensities of *B. thuringiensis*-specific BiDz, DNM3, and DNM4 in the absence or the presence of 10 ng of *B. thuringiensis* RNA after 60 min or 180 min of incubation at 55°C in the FAM channel. The data are average values of three independent measurements. The errors are given as one standard deviation of the average

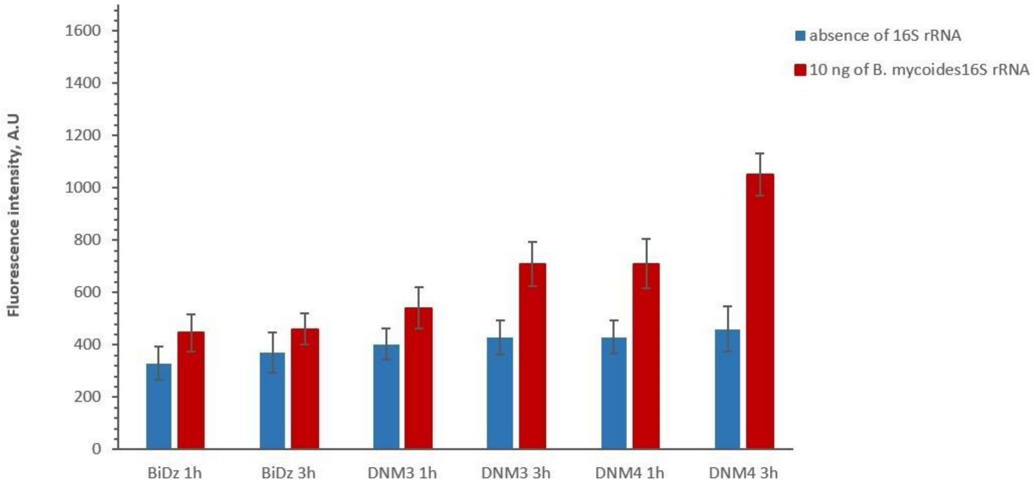

Figure S7. Comparison between the three DNA-nanosensors in detecting the bacterial *B. mycoides* 16S rRNA. Fluorescence intensities of *B. mycoides* -specific BiDz, DNM3, and DNM4 in the absence or the presence of 10 ng of *B. mycoides* RNA after 60 min or 180 min of incubation at 55°C in the Cy5 channel. The data are average values of three independent measurements. The errors are given as one standard deviation of the average.

8. Limit of the detection of B. thuringiensis-specific and B. mycoides-specific DNM1 for the total RNA.

A

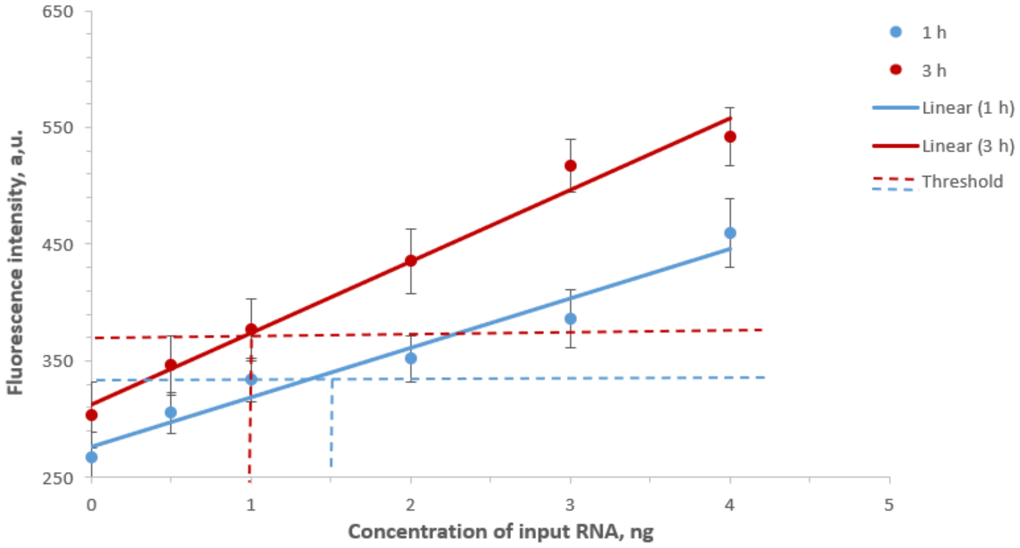

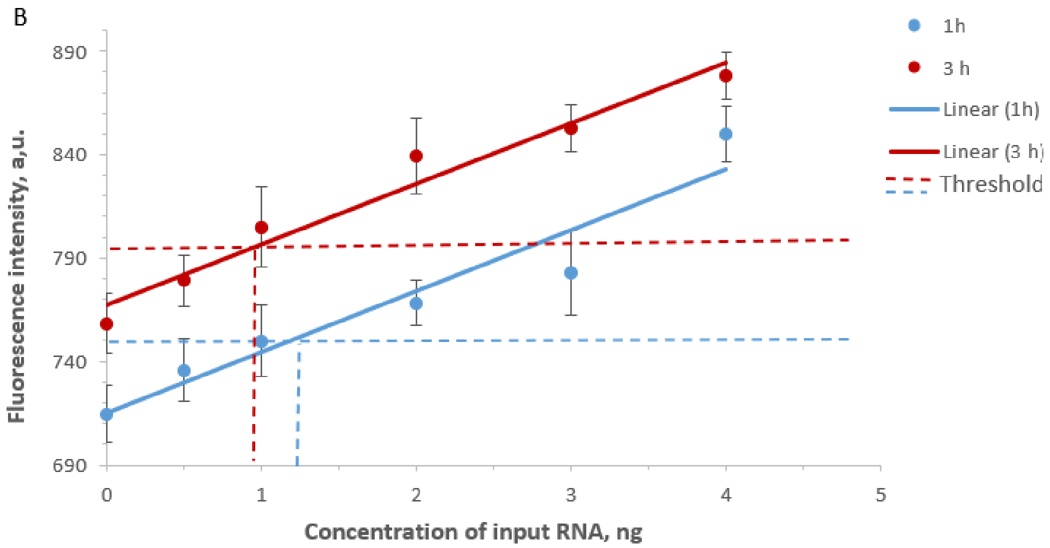

Figure S8. Limit of the detection of *B. thuringiensis*-specific and *B. mycoides*-specific DNM4 for the total RNA. A) Limit of the detection of *B. mycoides* - specific DNM4 for the RNA. Samples were incubated at 55°C in the reaction buffer (200mM $MgCl_2$, 150 mM KCl, 15 mM NaCl and 50 mM HEPES, pH 7.4) with different concentrations of the RNA (0-4 ng). B) Limit of the detection of *B. thuringiensis*- specific DNM4 for the RNA. Samples were incubated at 55°C in the reaction buffer with different concentrations of the RNA (0-4 ng). Fluorescent intensities were measured after 60 min and 180 min. The data are average values of three independent measurements. The errors are given as one standard deviation of the average. The Limit of detection is set as 3 standard deviations above the negative sample.

9. Limit of the detection of B. thuringiensis-specific and B. mycoides-specific DNM4 for the whole bacterial cells

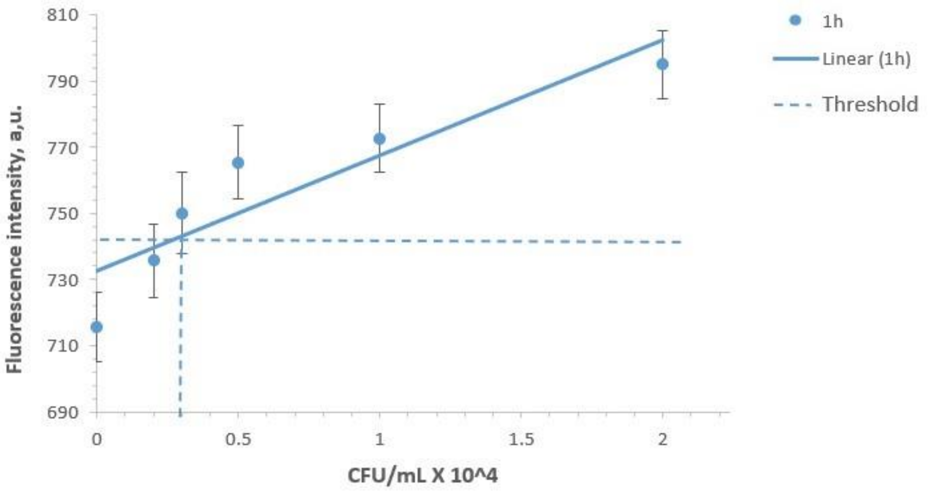

Figure S9. Limit of detection of the *B. thuringiensis*- specific DNM4 on the whole bacterial cells. Samples were incubated at 55°C in the reaction buffer (200mM $MgCl_2$, 150 mM KCl, 15 mM NaCl and 50 mM HEPES, pH 7.4) with different number of *B. thuringiensis* bacterial cells (0 - 2 x $10^{\wedge 4}$ CFU/mL). Fluorescent intensities were measured after 60 min in the FAM channel. The data are average values of three independent measurements. The errors are given as one standard deviation of the average. The Limit of detection is set as 3 standard deviations above the negative sample

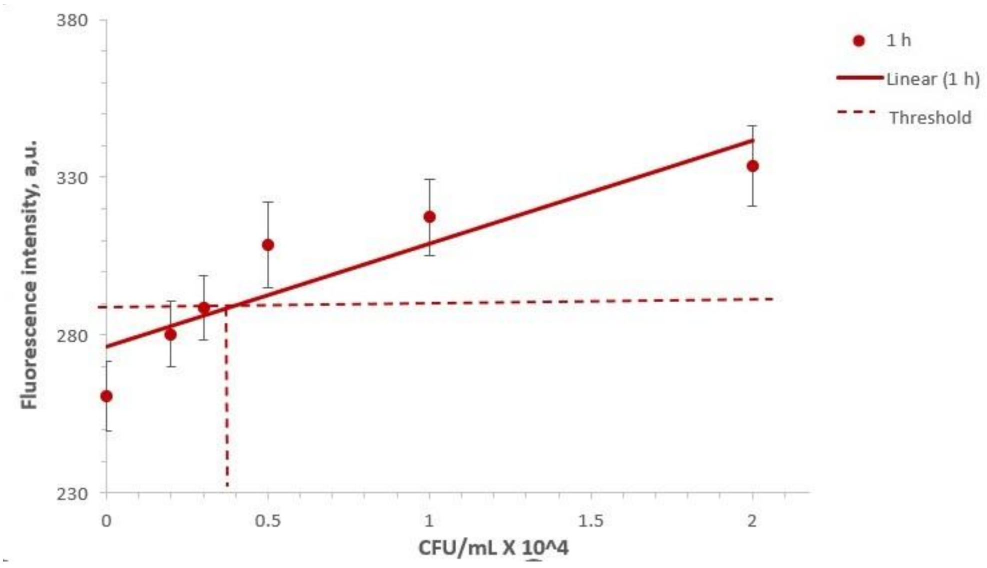

Figure S10. Limit of detection of the *B. mycoides* -specific DNM4 on the whole bacterial cells. Samples were incubated at 55°C in the reaction buffer (200mM MgCl2, 150 mM KCl, 15 mM NaCl and 50 mM HEPES, pH 7.4) with different number of *B. mycoides* bacterial cells (0- 2 x 10^4 CFU/mL). Fluorescent intensities were measured after 60 min in the Cy5 channel. The data are average values of three independent measurements. The errors are given as one standard deviation of the average. The Limit of detection is set as 3 standard deviations above the negative sample

10. Statistics

For the following results a signal was considered a true positive signal if it was higher than the background average plus three standard deviations as recommended [72].

Table S2. Data corresponding to Figure 3A in the main text.

Sensor name	Input concentration	0 pM	10 pM	50 pM	100 pM	200 pM	500 pM
4 DNM sensor	ΔF, a,u. for three repeats	0	60	85	120	122	164
		0	63	90	115	128	169
		0	59	97	111	130	157
	Average	0	61.3	90.6	115.3	126.6	163
3 DNM sensor	ΔF, a,u. for three repeats	0	40	72	90	108	127
		0	45	75	87	105	119
		0	38	69	94	100	121
	Average	0	41	72	90.3	104.3	122.3
BiDz sensor	ΔF, a,u. for three repeats	0	34	49	60	89	95
		0	26	43	67	80	108
		0	31	39	62	83	100
	Average	0	30	43	63	84	101

ΔF, a,u. was calculated by subtracting the background signal of the fluorophore and the sensor.

Table S3. Data corresponding to Figure 3B in the main text.

Sensor name	Input concentration	0 pM	10 pM	50 pM	100 pM	200 pM	500 pM
4 DNM sensor	ΔF, a,u. for three repeats	0	40	78	89	123	156
		0	45	70	98	113	150
		0	41	68	91	117	147
	Average	0	42	72	92.6	116.6	151
3 DNM sensor	ΔF, a,u. for three repeats	0	31	59	75	90	111
		0	25	49	77	95	120

		0	26	55	85	101	108
	Average	0	27.3	54.3	79	95.3	113
BiDz sensor	ΔF, a,u. for three repeats	0	23	38	59	70	98
		0	25	29	61	67	91
		0	18	34	56	77	87
	Average	0	22	33.6	58.3	71.3	92

ΔF, a,u. was calculated by subtracting the background signal of the fluorophore and the sensor.

Table S4. Data corresponding to Figure 4A in the main text.

Sensor name	Input	Blank	*B. thuringinesis* analyte	*B. mycoides* analyte	*B.mycodies* RNA	*B. cereus* RNA	*B. mycodies* cells	*B. cereus* cells	Cells spiked with SNS analyte
4DNM sensor	ΔF, a,u. for three repeats	34	125	35	37	31	33	38	95
		30	132	31	30	39	37	33	85
		25	127	38	33	34	38	35	88
	Average	29.6	128	34.6	33.3	34.6	36	35.3	89.3
BiDz sensor	ΔF, a,u. for three repeats	22	87	24	23	25	30	28	65
		27	81	30	29	28	28	31	60
		19	77	21	23	24	22	23	63
	Average	22.6	81.6	25	24.6	25.6	26.6	27.3	62.6

ΔF, a,u. was calculated by subtracting the average background signal of the fluorophore. A signal is considered a true positive signal if the ΔF, a,u is above ~ 43 for DNM4 sensor and above ~35 for BiDz sensor which corresponds to the blank average plus three standard deviations.

Table S5. Data corresponding to Figure 4B in the main text.

Sensor name	Input	Blank	*B. thuringinesis* analyte	*B. mycoides* analyte	*B.thuringiensis* RNA	*B. cereus* RNA	*B. thuringiensis* cells	*B. cereus* cells	Cells spiked with SNS analyte
4DNM sensor	ΔF, a,u. for three repeats	39	38	129	37	36	35	39	87
		34	40	118	40	35	41	42	78
		29	32	129	35	40	38	37	83
	Average	34	36.6	123.3	37.3	37.6	38	39.3	82.6
BiDz sensor	ΔF, a,u. for three repeats	31	35	88	34	31	38	34	55
		36	36	80	37	38	35	39	51
		28	30	85	35	34	32	33	58
	Average	31.6	33.6	84.3	35.3	34.3	35	35.3	54.6

ΔF, a,u. was calculated by subtracting the average background signal of the fluorophore. A signal is considered a true positive signal if the ΔF, a,u is above ~ 49 for DNM4 sensor and above ~44 for BiDz sensor which corresponds to the blank average plus three standard deviations.

Table S6. Data corresponding to Figure 5A in the main text.

Sensor name	Input	Blank	*B.thuringiensis* RNA	*B. mycodies* RNA	*B. cereus* RNA
4 DNM sensor	ΔF, a,u. for three repeats	36	77	39	34
		28	80	35	34
		31	84	31	37
	Average	31.6	80.3	35.3	34.6
BiDz sensor	ΔF, a,u. for three repeats	24	27	29	28
		27	28	22	25
		20	24	26	30
	Average	23.6	26	25.6	27.6

ΔF, a,u. was calculated by subtracting the average background signal of the fluorophore. A signal is considered a true positive signal if the ΔF, a,u is above ~ 43 for DNM4 sensor and above ~35 for BiDz sensor which corresponds to the blank average plus three standard deviations.

Table S7. Data corresponding to Figure 5B in the main text.

Sensor name	Input	Blank	*B.thuringiensis* RNA	*B. mycodies* RNA	*B. cereus* RNA
4 DNM sensor	ΔF, a,u. for three repeats	29	37	70	39
		32	34	68	37
		39	40	73	35
	Average	33.6	37	70.3	38
BiDz sensor	ΔF, a,u. for three repeats	32	38	36	39
		27	29	30	33
		35	34	34	35
	Average	31.3	33.6	33.3	35.6

ΔF, a,u. was calculated by subtracting the average background signal of the fluorophore. A signal is considered a true positive signal if the ΔF, a,u is above ~ 49 for DNM4 sensor and above ~43 for BiDz sensor which corresponds to the blank average plus three standard deviations.

Table S8. Data corresponding to Figure 6Ain the main text.

Sensor name	Input	Blank	2 × 10^4 *B. thuringiensis* cells	1.5 × 10^4 *B. thuringiensis* cells
4 DNM sensor	ΔF, a,u. for three repeats	29	89	66
		28	84	69
		35	80	61
	Average	30.6	80.4	65.3
BiDz sensor	ΔF, a,u. for three repeats	24	27	25
		23	31	29
		29	29	26
	Average	25.3	29	26.6

ΔF, a,u. was calculated by subtracting the average background signal of the fluorophore. A signal is considered a true positive signal if the ΔF, a,u is above ~ 43 for DNM4 sensor and above ~35 for BiDz sensor which corresponds to the blank average plus three standard deviations.

Table S9. Data corresponding to Figure 6B in the main text.

Sensor name	Input	Blank	2 × 10^4 *mycoides* cells	1.5 × 10^4 *B. mycoides* cells
4 DNM sensor	ΔF, a,u. for three repeats	29	72	63
		32	76	60
		39	69	58
	Average	33.6	72.3	60.3
BiDz sensor	ΔF, a,u. for three repeats	32	37	36
		27	31	29
		35	35	33
	Average	31.3	34.3	32.6

ΔF, a,u. was calculated by subtracting the average background signal of the fluorophore. A signal is considered a true positive signal if the ΔF, a,u is above ~ 49 for DNM4 sensor and above ~43 for BiDz sensor which corresponds to the blank average plus three standard deviations.

T-test was performed to determine if there is a significance difference between the average signal of *B. thuringiensis*-specific DNM4 and *B. mycoides*-specific DNM4 when it was tested on the target RNA or the whole cell with a comparison to the control strain of *B. cereus* or Blank. The p-value in all the tests were < 0.05.
